# Phylogeny predicts sensitivity in aquatic animals for only a minority of chemicals

**DOI:** 10.1007/s10646-024-02791-7

**Published:** 2024-07-22

**Authors:** Alice L. Coleman, Suzanne Edmands

**Affiliations:** University of Southern California Department of Biological Sciences, Los Angeles, CA USA

**Keywords:** Aquatic organisms, Chemical sensitivity, Cross-species extrapolation, Aquatic toxicology, Phylogenetics

## Abstract

There are substantial gaps in our empirical knowledge of the effects of chemical exposure on aquatic life that are unlikely to be filled by traditional laboratory toxicity testing alone. One possible alternative of generating new toxicity data is cross-species extrapolation (CSE), a statistical approach in which existing data are used to predict the effect of a chemical on untested species. Some CSE models use relatedness as a predictor of chemical sensitivity, but relatively little is known about how strongly shared evolutionary history influences sensitivity across all chemicals. To address this question, we conducted a survey of phylogenetic signal in the toxicity data from aquatic animal species for a large set of chemicals using a phylogeny inferred from taxonomy. Strong phylogenetic signal was present in just nine of thirty-six toxicity datasets, and there were no clear shared properties among those datasets with strong signal. Strong signal was rare even among chemicals specifically developed to target insects, meaning that these chemicals may be equally lethal to non-target taxa, including chordates. When signal was strong, distinct patterns of sensitivity were evident in the data, which may be informative when assembling toxicity datasets for regulatory use. Although strong signal does not appear to manifest in aquatic toxicity data for most chemicals, we encourage additional phylogenetic evaluations of toxicity data in order to guide the selection of CSE tools and as a means to explore the patterns of chemical sensitivity across the broad diversity of life.

## Introduction

Pollution represents one of the greatest threats to global biodiversity (Novacek and Cleland 2001). The biological consequences of pollution exposure are numerous, including reduced fecundity, compromised immune systems, developmental abnormalities and outright mortality (McKinlay et al. 2008), all of which threaten the health and functioning of organisms, populations and ecosystems at large. In recognition of this danger, regulatory bodies like the United States Environmental Protection Agency (EPA) manage pollution in part by setting numeric criteria that are intended to protect life by limiting the accumulation of hazardous concentrations of chemicals in the environment. The development of such criteria depends on empirical data that describe the concentrations of chemicals that inflict adverse effects on organisms, however, most chemicals do not have robust toxicity datasets (Wheeler et al. 2002; Dowse et al. 2013; Coleman and Edmands 2022).

Most toxicity data are derived from single-species toxicity tests that measure the dose of a substance that induces adverse effects in a test population over a certain period. These tests are classified as either acute (short-term) or chronic (long-term), with chronic tests typically performed less frequently because of the high costs associated with long-term testing. In theory, the species evaluated in such tests should be representative of the biological community a criterion is intended to protect, but in practice, most testing is performed using a select group of model species (Seegert et al. 1985; Anderson and Phillips 2016; Buchwalter et al. 2017). As a result, our existing toxicity database does not necessarily reflect the broad diversity of life nor the entire spectrum of sensitivity to any given chemical. The logical means of addressing these data gaps would be to simply increase testing, but the sheer number of possible species-chemical combinations means that expanding laboratory efforts is not a viable solution. As a result, there is a demand for other tools that can reliably estimate how sensitive a species is to any given chemical. At present, computational methods that extrapolate existing toxicity data to untested species (i.e cross-species extrapolation) represent the most promising alternative to traditional laboratory testing (van den Berg et al. 2021; LaLone et al. 2021).

There are four main types of cross-species extrapolation (CSE) models: interspecies correlation, trait, genomic and relatedness-based (van den Berg et al. 2021). Here, we are most interested in relatedness-based models, which operate under the assumption that evolutionary relationships can explain the variation in chemical sensitivity across taxa. These models generate new toxicity data for species by using metrics of relatedness as predictors of sensitivity. One such metric used in relatedness-based CSE is phylogenetic signal, which is a measure of the statistical dependence among species trait values that arises from their phylogenetic relationships (Revell et al. 2008; van den Berg et al. 2021). When phylogenetic signal is high, close relatives on a phylogeny will tend to exhibit very similar trait values while distantly related species will not. When signal is low, trait values will tend to be randomly distributed across a phylogeny and distantly related taxa may resemble each other more than close relatives (Kamilar and Muldoon 2010; Kamilar and Cooper 2013). Chemical sensitivity is regarded as a possible candidate to exhibit strong phylogenetic signal (Hylton et al. 2018).

Sensitivity to a chemical is a complex phenotype determined by a network of interacting morphological, physiological and behavioral traits that regulate the uptake, metabolism and excretion of pollutants by an organism. Many traits linked to sensitivity such as body size and metabolic rate are known to exhibit phylogenetic signal, suggesting that we are likely to find some amount of signal in chemical sensitivity data (Kamilar and Cooper 2013; Hylton et al. 2018). To date, only a small number of studies have evaluated toxicity data for phylogenetic signal, some of which identified meaningful associations between shared evolutionary history and species sensitivity (Buchwalter et al. 2008; Guénard et al. 2011; Hammond et al. 2012; Chiari et al. 2015; Hylton et al. 2018; Moore et al. 2020; Duque et al. 2023). The scope of this work has been relatively narrow to date, with emphasis placed on data from either a single chemical or taxonomic group at a time. These limitations mean that we do not have a full understanding of the abundance of strong phylogenetic signal in toxicity data and so the practicality of using relatedness-based CSE to fill data gaps on a large scale remains in question.

Improved knowledge of phylogenetic signal in toxicity data may have other uses aside from cross-species extrapolation. One option would be to test hypotheses that explain why phylogenetic signal manifests more strongly in some datasets than others. To this end, we propose three hypotheses based on qualitative properties of chemicals and toxicity data. First, we predict that strong phylogenetic signal will be more common among synthetic chemicals than naturally occurring chemicals. Synthetic chemicals are relative newcomers in the environment, so potentially only a subset of lineages will have had enough exposure for selection to favor increased tolerance. Second, we predict that strong phylogenetic signal will be more common in the data from chemicals with specific toxic modes of action (MOAs) than those that are nonspecific. This is because specific MOAs interfere with biological processes by precisely binding to a particular site or molecule, so an adaptive phenotype could in theory arise after only a small number of molecular changes (Whitehead et al. 2017; Gupta 2018). Chemicals with nonspecific MOAs tend to induce generic stress responses, so the corresponding adaptive phenotypes may be complex and thus take much longer to develop (Whitehead et al. 2017). Lastly, we predict that strong phylogenetic signal will be more common in chronic toxicity datasets than acute because rapid responses to acute stress are often generalized and evolutionarily conserved (Kültz 2020). In contrast, responses to chronic stress have been found to be more chemical- and lineage-specific (Kovalchuk et al. 2007; McRae et al. 2022). Testing hypotheses such as these may provide insight into whether qualitative properties are viable predictors of strong phylogenetic signal, which would be useful for identifying the datasets to which relatedness-based CSE models can be applied.

In this study we expand upon previous phylogenetic analyses in ecotoxicology by considering toxicity data from both a large number of chemical pollutants and a biologically diverse set of species. Using published laboratory toxicity data and a phylogeny inferred from taxonomy, we quantified the phylogenetic signal present in these data and investigated whether various chemical properties and experimental conditions such as temperature and pH affected phylogenetic signal. When strong signal was identified, we additionally used the phylogenetic analyses to identify the sensitive and resistant clades within a dataset. The application of CSE methods to environmental risk assessment and regulation has been a recent focus in aquatic toxicology (Raimondo et al. 2010; Schlekat et al. 2010; Lewis and Thursby 2018; Coleman and Edmands 2022), so here our analysis specifically dealt with data similar to those used in water quality criteria development.

## Methods

### Data Collection

Toxicity data from aquatic species were collected for twenty-four chemicals (Table 1) that span a range of classes and modes of action (MOA). The MOA of each chemical was obtained and labeled as either generic or precise using the MOAtox database from Barron et al. (2015). Acute toxicity data were then obtained from the EPA’s ECOTOXicology Knowledgebase (ECOTOX; Olker et al. 2022) using these search parameters:

1. Chemical Abstracts Service (CAS) Registry Number
2. Endpoint: Median Lethal Concentration (LC50)
3. Kingdom: Animals
4. Test Location: Lab
5. Exposure Media: Freshwater or Saltwater
6. Duration: 48 or 96 h

**Table 1:** Properties of chemicals evaluated in analyses

Chemical	CAS RN	Origin	Class	Mode of Action
Ammonia	7664-41-7	Natural	Inorganic	Osmoregulatory impairment^a^
Cadmium	7440-43-9	Natural	Metal	Metallic iono/osmoregulatory impairment^a^
Chlorine	7782-50-5	Natural	Halogen	Osmoregulatory impairment^a^
Copper	7440-50-8	Natural	Metal	Metallic iono/osmoregulatory impairment^a^
Mercury	7439-97-6	Natural	Metal	Metallic iono/osmoregulatory impairment^a^
Nickel	7440-02-0	Natural	Metal	Metallic iono/osmoregulatory impairment^a^
Phenol	108-95-2	Natural	Phenol	Polar narcosis^a^
Toluene	108-88-3	Natural	Aromatic Hydrocarbon	Nonpolar narcosis^a^
Zinc	7440-66-6	Natural	Metal	Metallic iono/osmoregulatory impairment^a^
4-Nitrophenol	100-02-7	Synthetic	Nitrophenol	Polar narcosis^a^
Atrazine	1912-24-9	Synthetic	Triazine	Narcosis^a^
Chlorpyrifos	2921-88-2	Synthetic	Organophosphate	AChE Inhibition^b^
DDT	50-29-3	Synthetic	Organochlorine	Neurotoxicity^b^
Diazinon	333-41-5	Synthetic	Organophosphate	AChE inhibition^b^
Dieldrin	60-57-1	Synthetic	Organochlorine	Neurotoxicity^b^
Endosulfan	115-29-7	Synthetic	Organochlorine	Neurotoxicity^b^
Endrin	72-20-8	Synthetic	Organochlorine	Neurotoxicity^b^
Glyphosate	1071-83-6	Synthetic	Phosphonate	Nonpolar narcosis^a^
Guthion	86-50-0	Synthetic	Organophosphate	AChE inhibition^b^
Lindane	58-89-9	Synthetic	Organochlorine	Neurotoxicity^b^
Malathion	121-75-5	Synthetic	Organophosphate	AChE inhibition^b^
Parathion	56-38-2	Synthetic	Organophosphate	AChE inhibition^b^
PCP	87-86-5	Synthetic	Organochlorine	Electron transport inhibition^b^
TBTO	56-35-9	Synthetic	Organotin	Electron transport inhibition^b^

*CAS RN* Chemical Abstracts Service Registry Number, *DDT* 1,1′-(2,2,2-Trichloroethane-1,1-diyl)bis(4-chlorobenzene), *PCP* Pentachlorophenol, *TBTO* Tributyltin oxide, *AChE* Acetylcholinesterase

^a^Generic mode of action

^b^Precise mode of action

An LC50 represents the concentration of a toxic substance that kills half of the test organisms during the testing period. LC50s from 96 h tests were used for all animals except cladocerans and midges, for which we collected 48 h data. These parameters were specifically designed to approximate the data collection rules stated in the EPA’s methodology for water quality criteria development (Stephan et al. 1985).

Chronic toxicity data were similarly mined from ECOTOX for twelve chemicals (Table 2) using the following parameters:

1. CAS Registry Number
2. Endpoint: No Observed Effect Concentration (NOEC)
3. Kingdom: Animals
4. Test Location: Lab
5. Exposure Media: Freshwater or Saltwater
6. Duration: 7 – 60 days

**Table 2:** Phylogenetic signal results for all dataset variations

Chemical	Exposure	Dataset Variation				
		Complete	Subadult	Temperature	pH	Hardness
		λ	λ	λ	λ	λ
Ammonia	Acute	7.30E-05	7.40E-05	**0.95**	1.00E-06	-
Atrazine	Acute	7.30E-05	7.30E-05	1.00E-06	1.00E-06	1.00E-06
Atrazine	Chronic	7.30E-05	7.30E-05	1.00E-06	**0.57**	**0.92**
Cadmium	Acute	7.30E-05	0.28	1.00E-06	0.029	-
Cadmium	Chronic	**1**	**1**	1.00E-06	1.00E-06	-
Chlorine	Acute	**0.9**	**0.69**	1.00E-06	1.00E-06	**0.99**
Chlorpyrifos	Acute	7.30E-05	7.30E-05	1.00E-06	1.00E-06	-
Chlorpyrifos	Chronic	7.30E-05	0.23	0.025	0.029	-
Copper	Acute	7.30E-05	**0.72**	0.27	0.13	-
Copper	Chronic	**1**	7.40E-05	1.00E-06	1.00E-06	-
DDT	Acute	0.00065	**1**	1.00E-06	1.00E-06	-
Diazinon	Acute	0.023	0.025	**0.92**	**0.81**	-
Diazinon	Chronic	**1**	**1**	1.00E-06	1.00E-06	-
Dieldrin	Acute	7.30E-05	0.0028	1.00E-06	1.00E-06	-
Endosulfan	Acute	0.03	7.30E-05	1.00E-06	0.077	-
Endosulfan	Chronic	0.11	0.18	1.00E-06	1.00E-06	-
Endrin	Acute	7.30E-05	7.30E-05	**0.58**	**0.59**	-
Glyphosate	Acute	7.40E-05	**0.82**	1.00E-06	1.00E-06	-
Glyphosate	Chronic	7.30E-05	7.30E-05	0.29	0.32	-
Guthion	Acute	**0.56**	0.39	1.00E-06	0.028	0.069
Lindane	Acute	**1**	**0.84**	0.17	**0.99**	-
Lindane	Chronic	**1**	7.50E-05	1.00E-06	1.00E-06	0.019
Malathion	Acute	0.35	0.37	1.00E-06	1.00E-06	-
Malathion	Chronic	7.30E-05	7.40E-05	0.029	**1**	-
Mercury	Acute	0.16	7.40E-05	1.00E-06	0.27	**1**
Nickel	Acute	7.30E-05	0.16	1.00E-06	1.00E-06	-
Nitrophenol	Acute	7.40E-05	7.40E-05	1.00E-06	**1**	**1**
Parathion	Acute	0.16	**0.5**	1.00E-06	1.00E-06	-
PCP	Acute	**0.99**	**0.58**	**0.63**	**0.74**	-
PCP	Chronic	7.40E-05	7.40E-05	0.1	0.086	-
Phenol	Acute	0.33	7.40E-05	1.00E-06	1.00E-06	-
Phenol	Chronic	**1**	**0.9**	1.00E-06	1.00E-06	-
TBTO	Acute	7.30E-05	7.40E-05	1.00E-06	0.24	-
Toluene	Acute	7.40E-05	**0.96**	1.00E-06	1.00E-06	-
Zinc	Acute	0.033	7.40E-05	**1**	1.00E-06	-
Zinc	Chronic	7.30E-05	0.38	1.00E-06	**1**	**1**

*DDT* 1,1′-(2,2,2-Trichloroethane-1,1-diyl)bis(4-chlorobenzene), *PCP* Pentachlorophenol, *TBTO* Tributyltin oxide

Values in boldface indicate strong phylogenetic signal (λ > 0.5

The NOEC represents the highest concentration of a chemical that does not induce a response that differs significantly from the control treatment in a toxicity test. The remaining 12 chemicals from the initial set of 24 were excluded because they did not have NOEC data from at least ten unique species available in ECOTOX. As there is no universally agreed upon period that constitutes a chronic toxicity test, we allowed for a broad range of durations to maximize the amount of usable data.

Test organism life stage, experimental temperature, pH and hardness (for metals only) for each toxicity value were also obtained from ECOTOX when possible because these variables are known to modify the toxicity of many chemicals to aquatic species (Cairns et al. 1975; Yim et al. 2006; Pinheiro et al. 2021; Kazmi et al. 2022). Organism life stage was standardized to either “subadult”, “adult” or “NR” (not reported) based on the information provided by ECOTOX. We then filtered the data based on the availability of each experimental variable to create the following five variants of each dataset:

1. Complete: all toxicity values obtained from ECOTOX
2. Subadult: only toxicity values derived from subadult test organisms
3. Temperature: only toxicity values with experimental temperature information
4. pH: only toxicity values with experimental pH information
5. Hardness: only toxicity values with experimental hardness information

If more than one toxicity value was available for a given chemical-species pair, the values were summarized as their geometric mean. Similarly, if the toxicity values for a chemical-species pair were derived from tests performed at different temperatures, pH or hardnesses, information from each variable was summarized as a geometric mean.

### Phylogenetic analyses

We used the National Center for Biotechnology Information’s (NCBI) Taxonomy database via the phyloT generator (Letunica 2022) to produce a single comprehensive phylogenetic tree for all of the species that appeared in the toxicity datasets we assembled. Species without a matching entry in the NCBI database were excluded from the tree. We imported the tree generated by phyloT into R using the package ape (Paradis and Schliep 2019), where we then computed its branch lengths using Grafen’s transformation (*ρ* = 1; Grafen and Hamilton 1989) and annotated its tips with the toxicity data.

**Fig. 1:**
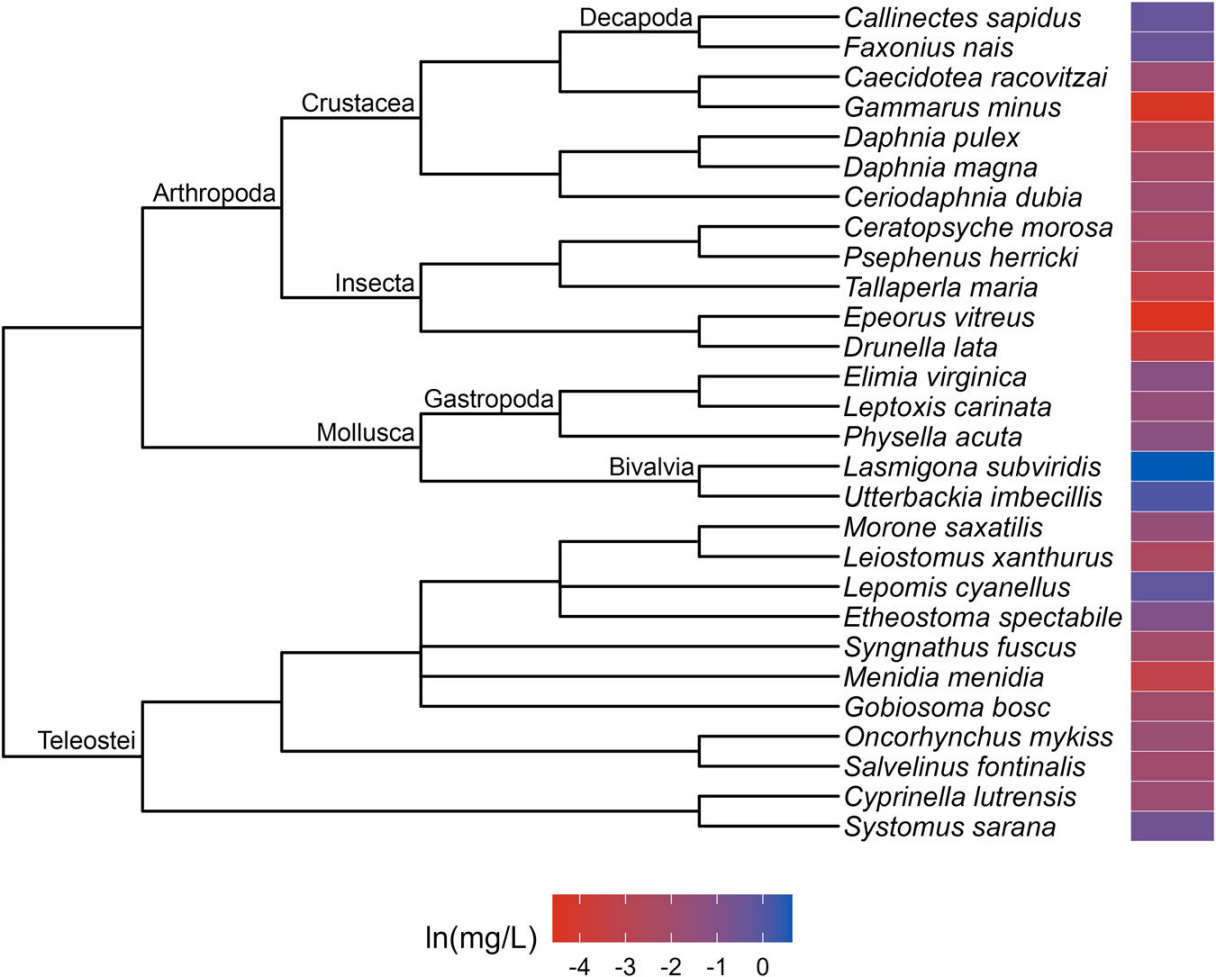
Phylogenetic tree and toxicity data heatmap for the acute chlorine dataset (λ = 0.9). The colored bar next to each species represents its relative sensitivity to the chemical. A red bar indicates a high degree of sensitivity (i.e. small amount of chemical causes toxic effect), while a blue bar indicates low sensitivity (i.e. large amount of chemical causes toxic effect)

**Fig. 2:**
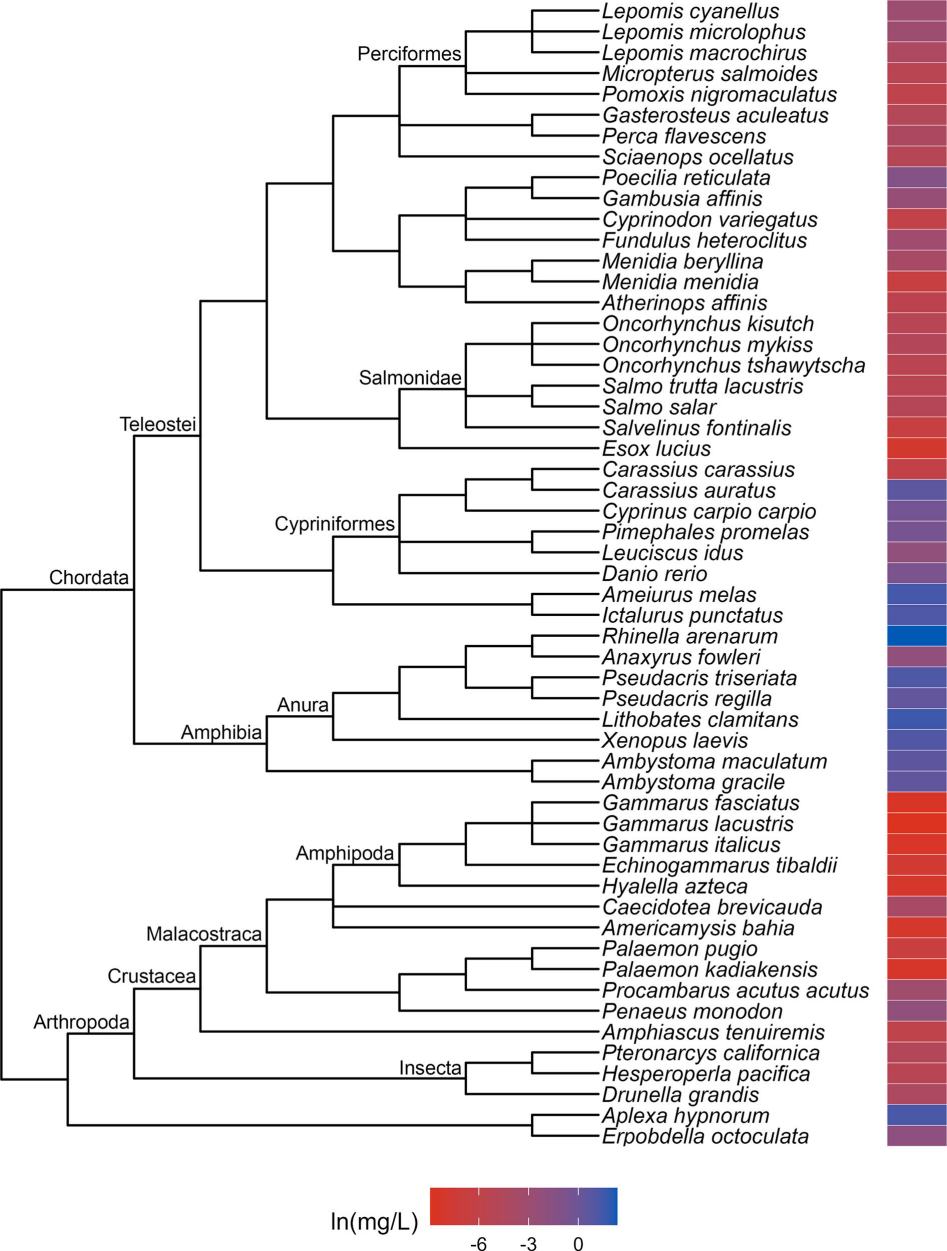
Phylogenetic tree and toxicity data heatmap for the acute guthion dataset (λ = 0.56). The colored bar next to each species represents its relative sensitivity to the chemical. A red bar indicates a high degree of sensitivity (i.e. small amount of chemical causes toxic effect), while a blue bar indicates low sensitivity (i.e. large amount of chemical causes toxic effect)

**Fig. 3:**
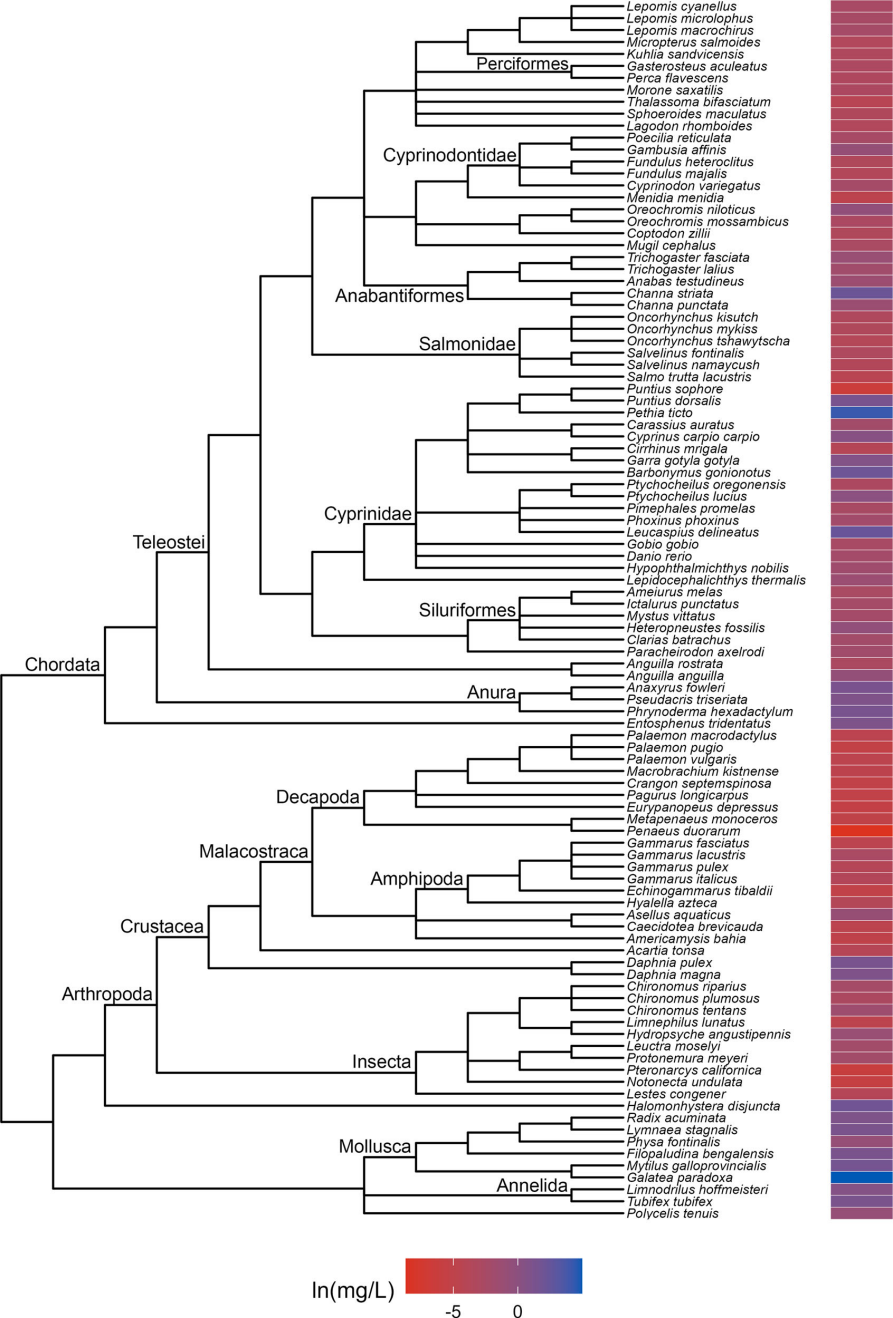
Phylogenetic tree and toxicity data heatmap for the acute lindane dataset (λ = 1). The colored bar next to each species represents its relative sensitivity to the chemical. A red bar indicates a high degree of sensitivity (i.e. small amount of chemical causes toxic effect), while a blue bar indicates low sensitivity (i.e. large amount of chemical causes toxic effect)

**Fig. 4:**
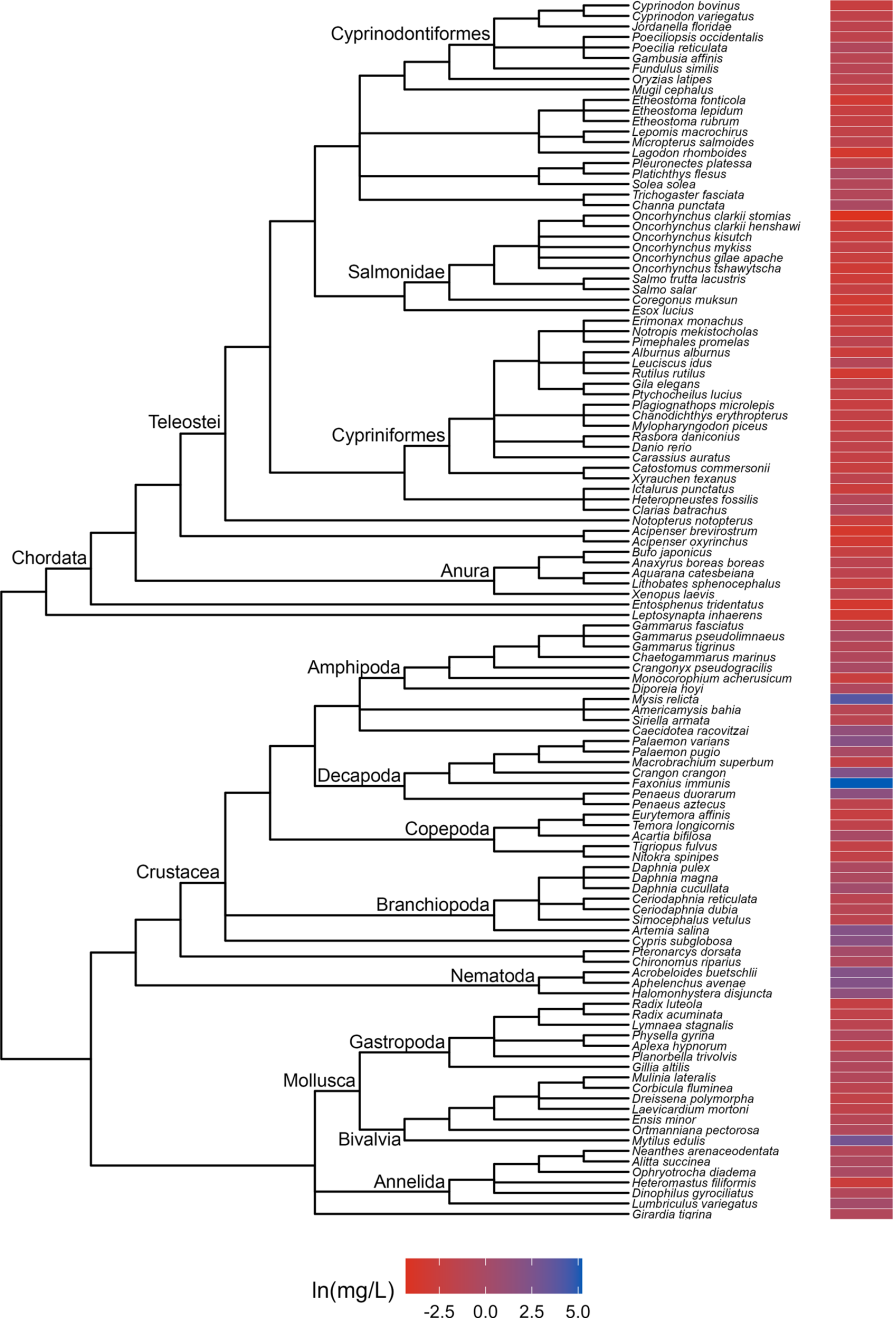
Phylogenetic tree and toxicity data heatmap for the acute pentachlorophenol dataset (λ = 0.99). The colored bar next to each species represents its relative sensitivity to the chemical. A red bar indicates a high degree of sensitivity (i.e. small amount of chemical causes toxic effect), while a blue bar indicates low sensitivity (i.e. large amount of chemical causes toxic effect)

**Fig. 5:**
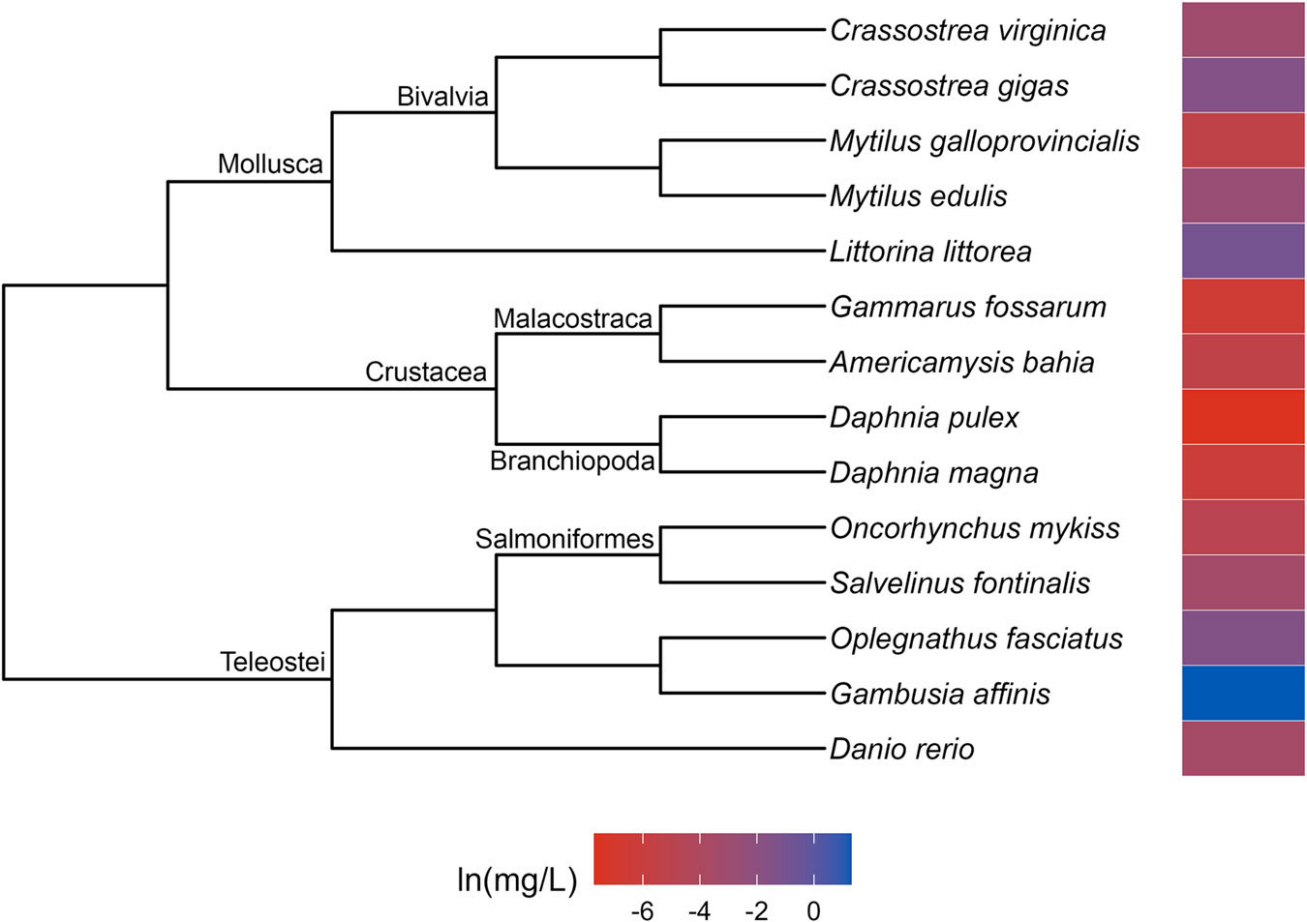
Combined phylogenetic tree and toxicity data heatmap for the chronic cadmium dataset (λ = 1). The colored bar next to each species represents its relative sensitivity to the chemical. A red bar indicates a high degree of sensitivity (i.e. small amount of chemical causes toxic effect), while a blue bar indicates low sensitivity (i.e. large amount of chemical causes toxic effect)

**Fig. 6:**
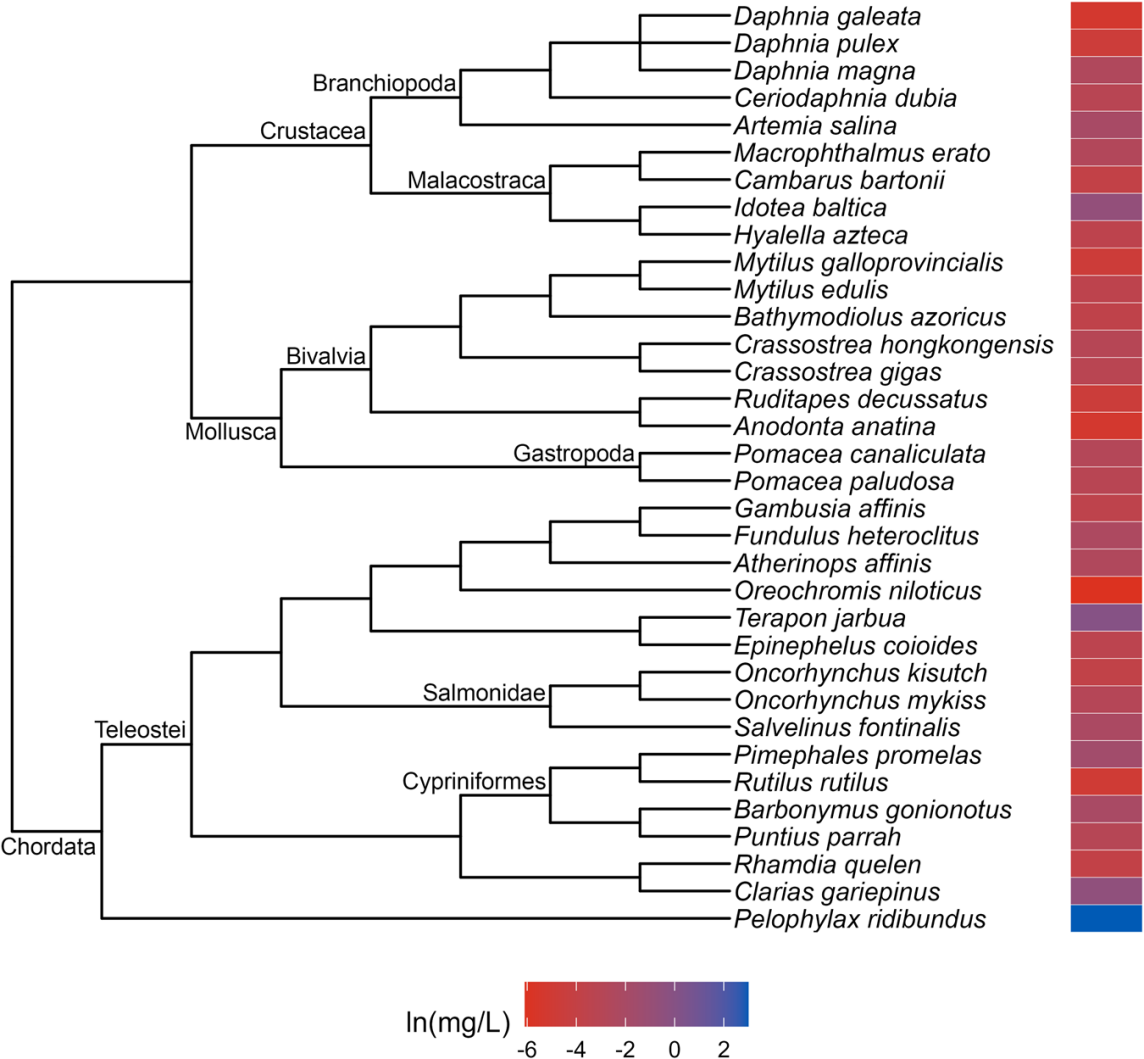
Combined phylogenetic tree and toxicity data heatmap for the chronic copper dataset (λ = 1). The colored bar next to each species represents its relative sensitivity to the chemical. A red bar indicates a high degree of sensitivity (i.e. small amount of chemical causes toxic effect), while a blue bar indicates low sensitivity (i.e. large amount of chemical causes toxic effect)

**Fig. 7:**
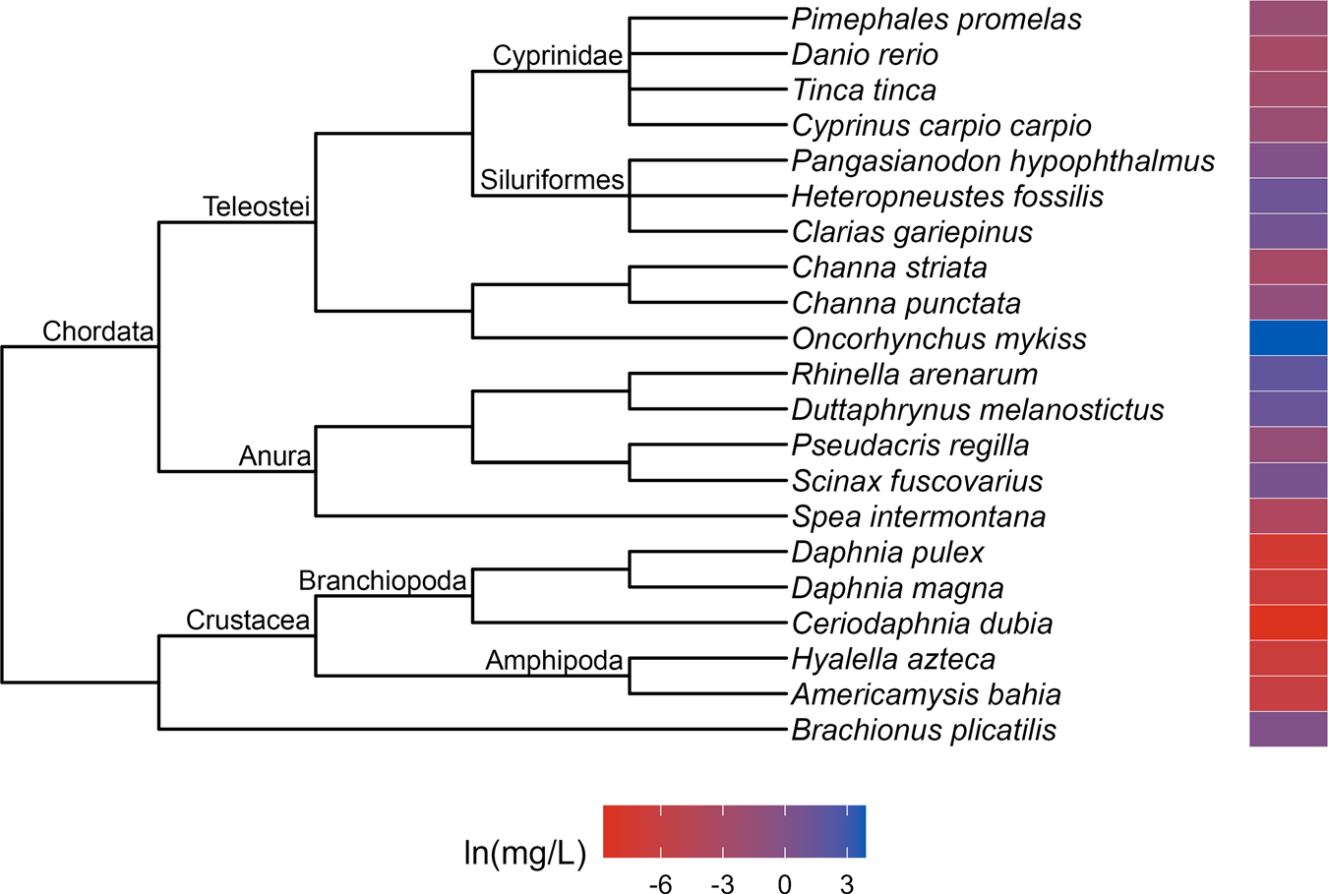
Combined phylogenetic tree and toxicity data heatmap for the chronic diazinon dataset (λ = 1). The colored bar next to each species represents its relative sensitivity to the chemical. A red bar indicates a high degree of sensitivity (i.e. small amount of chemical causes toxic effect), while a blue bar indicates low sensitivity (i.e. large amount of chemical causes toxic effect)

**Fig. 8:**
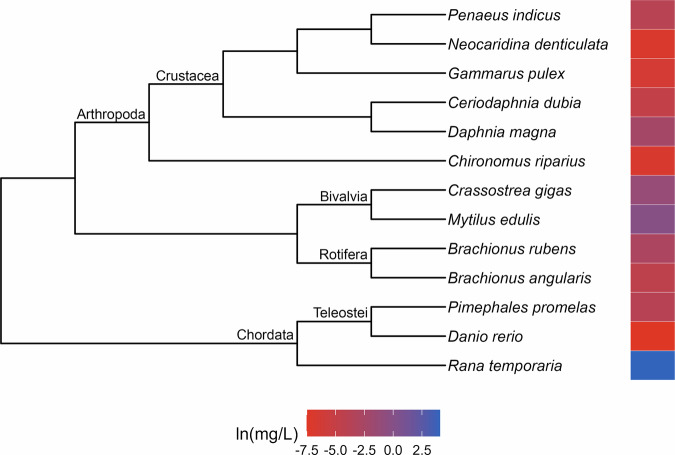
Combined phylogenetic tree and toxicity data heatmap for the chronic lindane dataset (λ = 1). The colored bar next to each species represents its relative sensitivity to the chemical. A red bar indicates a high degree of sensitivity (i.e. small amount of chemical causes toxic effect), while a blue bar indicates low sensitivity (i.e. large amount of chemical causes toxic effect)

**Fig. 9:**
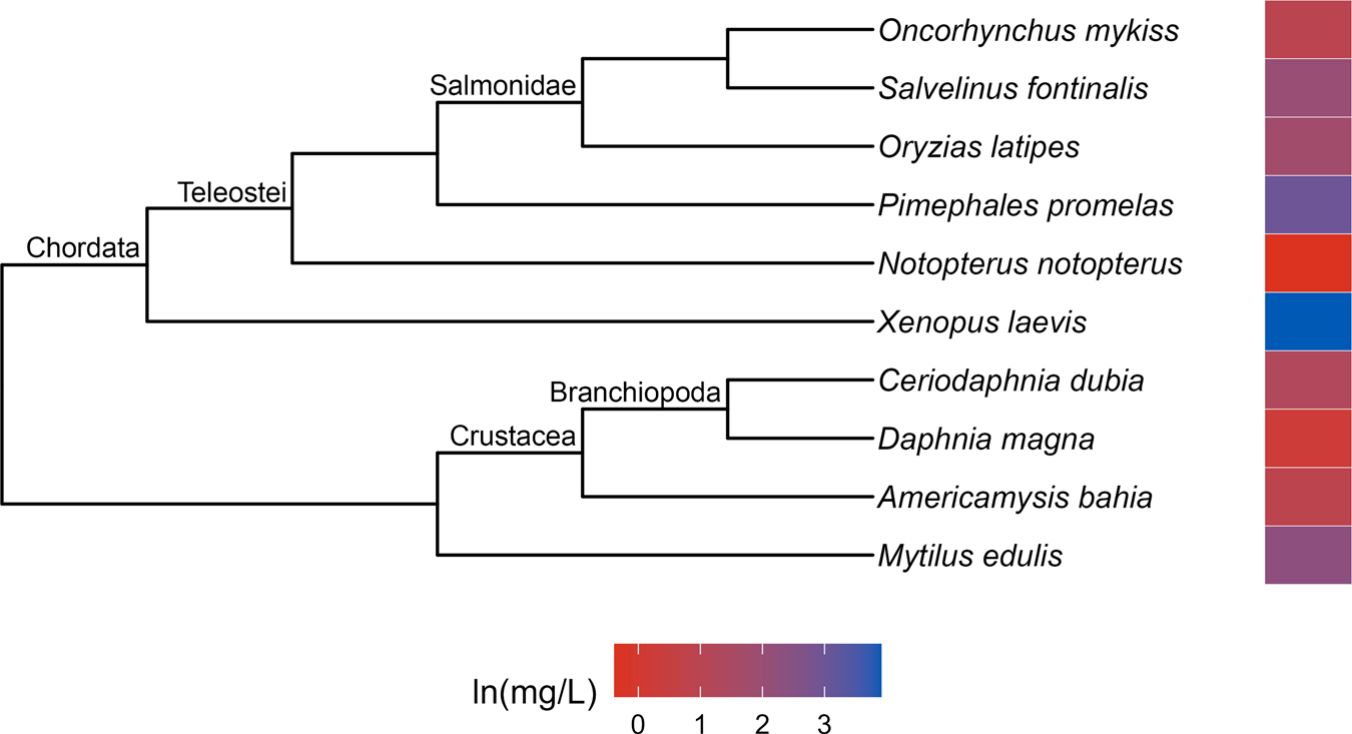
Combined phylogenetic tree and toxicity data heatmap for the chronic phenol dataset (λ = 1). The colored bar next to each species represents its relative sensitivity to the chemical. A red bar indicates a high degree of sensitivity (i.e. small amount of chemical causes toxic effect), while a blue bar indicates low sensitivity (i.e. large amount of chemical causes toxic effect)

Phylogenetic signal was estimated as Pagel’s λ (Pagel 1998). The values of λ range between zero and one, where a value close to zero indicates that the trait is phylogenetically independent and a λ near one indicates that the distribution of the trait is consistent with the Brownian Motion model, wherein closely related lineages are more similar to each other than those that are distantly related (Münkemüller et al. 2012). Following Hylton et al. (2018), we considered λ values of 0.5 or greater to be evidence of strong phylogenetic signal. A maximum of five calculations of λ were performed for each toxicity dataset and its corresponding variants. The first two were conducted using the function phylosig from the R package phytools (Revell 2012) for the complete dataset while the second was performed for the subset of toxicity data that were derived from subadult test organisms. When strong signal was identified in a complete dataset, we plotted the toxicity data alongside the phylogeny (Figs. 1–9) and used it to identify taxonomic patterns of sensitivity. We used the second calculation of λ to determine whether the frequency of strong phylogenetic signal in the toxicity data differed in organisms tested at early (subadult) versus various (subadult, adult and NR) life stages.

Calculations of λ for the temperature, pH and hardness dataset variants were evaluated using linear phylogenetic generalized least squares (PGLS; Grafen and Hamilton 1989) models. A form of regression analysis, PGLS models are typically used to test the association between variables while statistically controlling for the effects of phylogenetic signal in the data (Symonds and Blomberg 2014). These models also include a measurement of λ in a dataset which is calculated on the model’s residuals using maximum likelihood estimation (Revell 2010; Symonds and Blomberg 2014; Pagel 1999). Following Chiari et al. (2015) and Hylton et al. (2018), we used PGLS to calculate phylogenetic signal while statistically controlling for variation in experimental conditions. PGLS models were implemented using the package caper (Orme et al. 2018; Grafen and Hamilton 1989), in which toxicity (LC50 or NOEC) was set as the dependent variable and the environmental information (temperature, pH or hardness) as the predictor variable. Temperature, pH and hardness were modeled separately because most toxicity values did not have information for all three variables available.

## Results

### Data composition

We collected a cumulative total of 9138 datapoints to create 36 toxicity datasets (24 acute, 12 chronic) for 24 chemicals (9 natural, 15 synthetic; 12 generic MOAs, 12 precise MOAs). Together, these datasets included values from 595 unique species from ten phyla (Annelida, Arthropoda, Bryozoa, Chordata, Cnidaria, Echinodermata, Mollusca, Nematoda, Platyhelminthes and Rotifera). The sizes of the toxicity datasets varied considerably, with the largest (acute chlorpyrifos) composed of 993 toxicity values from 112 species while the smallest (chronic lindane) was made up of just 33 toxicity values from 13 species. In general, acute toxicity datasets were larger than chronic and synthetic chemical datasets were larger those of the naturally-occurring chemicals (Table S1). Dataset sample sizes decreased substantially when filtered by availability of life stage, temperature, pH or hardness information (Table S1).

### Phylogenetic signal

Strong phylogenetic signal (λ ≥ 0.5) was identified in nine of the 36 complete toxicity datasets (acute chlorine, acute guthion, acute lindane, acute pentachlorophenol, chronic cadmium, chronic copper, chronic diazinon, chronic lindane and chronic phenol) when we did not consider any of life stage, experimental temperature, pH or hardness (Table 2). Convergence on the lower bound of λ was common, as 18 datasets returned a signal value at or near 7.3E-05. Fisher’s exact tests indicated that there was no difference in frequencies of strong phylogenetic signal between complete toxicity datasets from chemicals of different origins (natural vs. synthetic; *p* = 0.68), classes (see “Class” column in Table 1; *p* = 0.86) or modes of action (generic vs. precise; *p* = 1). Similarly, the frequencies of strong phylogenetic signal in acute and chronic toxicity data were the same (*p* = 0.085).

Strong phylogenetic signal was also identified in eleven of 36 subadult, five of 36 temperature, eight of 36 pH and five of eight hardness datasets However, the frequencies of strong signal in each of these dataset variants were not significantly different from the frequency of strong signal in the complete datasets according to comparisons performed with Fisher’s exact tests (complete vs. subadult *p* = 0.79; complete vs. temperature *p* = 0.37, complete vs. pH *p* = 1, complete vs. hardness *p* = 1).

### Patterns of sensitivity

The patterns of relative sensitivity in the complete acute toxicity datasets with strong phylogenetic signal varied across chemicals. For example, the data for chlorine (λ = 0.9; Fig. 1) suggests that insects are highly sensitive while fishes, crustaceans and molluscs are more tolerant. Within the crustaceans, the tolerance of decapods appears to exceed that of the amphipods while in the molluscs, bivalves are more resistant than gastropods. The guthion dataset (λ = 0.56; Fig. 2) indicates that amphibians and Cypriniform fishes are resistant to guthion exposure, while the crustaceans and fishes from the order Perciformes and family Salmonidae are highly sensitive. Among the taxa with acute lindane data (λ = 1; Fig. 3), the decapods and amphipods appear to be very sensitive while the molluscs, annelids and frog species are relatively more resistant, while the acute pentachlorophenol dataset (λ = 1; Fig. 4) suggests that most species are highly vulnerable during short-term exposure to the organochlorine pesticide.

The patterns of relative sensitivity were similarly variable in the chronic toxicity datasets. The cadmium data (λ = 1; Fig. 5) indicates that crustaceans (Malacostraca and Branchiopoda) are highly sensitive to long-term exposure to the metal while molluscs and fishes are more tolerant. The majority of species featured in the chronic copper dataset (λ = 1; Fig. 6) seem to experience toxic effects when exposed to low levels of copper, with high relative tolerance evident in just one species of frog (*Pelophylax ridibundus*) and a few cases of moderate tolerance scattered across different clades. Chordates largely appear to be tolerant of chronic diazinon exposure (λ = 1; Fig. 7), while crustaceans are more sensitive. Crustaceans also appear highly vulnerable to chronic lindane exposure (λ = 1; Fig. 8), while bivalves exhibit relatively moderate tolerance and low sensitivity only evident in one frog species (*Rana temporaria*). Similarly, a frog species (*Xenopus laevis*) was the only taxon to exhibit relatively high resistance to phenol while the crustaceans seem to be more sensitive (λ = 1; Fig. 9).

The phylogenetic trees for datasets without strong phylogenetic signal (λ < 0.5) are provided in the Supplementary Information (Figs. S1–S27).

### PGLS

Experimental temperature, pH and hardness were significant predictors of toxicity in PGLS models for six, two and two datasets each respectively (Table 3). The adjusted R^2^ values of the significant models ranged between 0.04 and 0.47 for temperature, between 0.26 and 0.79 for pH and between 0.94 and 1 for hardness (Table 3). Experimental temperature was positively associated with tolerance (i.e. LC50/NOEC increases when temperature increases) in two datasets and negatively associated with tolerance (i.e. LC50/NOEC decreases when temperature increases) in the other four with significant models. Experimental pH was positively associated with tolerance in the acute chlorine data and negatively associated with tolerance in the chronic zinc data. Hardness was positively associated with tolerance of acute chlorine exposure, and negatively associated with acute mercury tolerance.

**Table 3:** Results of PGLS regressions for temperature and pH with toxicity data

Chemical	Exposure	Dataset Variation								
		Temperature			pH			Hardness		
		Coef	*p*	R^2^	Coef	*p*	R^2^	Coef	*p*	R^2^
Ammonia	Acute	**8**	**0.003**	**0.19**	−53	0.14	0.033	-	-	-
Atrazine	Acute	1.1	0.75	−0.02	1.9	0.82	−0.02	0.03	0.3	0.006
Atrazine	Chronic	0.045	0.76	−0.01	−0.05	0.97	−0.02	0.008	0.12	0.04
Cadmium	Acute	−***2.3***	***0.018***	***0.075***	−1.5	0.95	−0.02	-	-	-
Cadmium	Chronic	−0.01	0.47	−0.05	−0.02	0.39	0.006	-	-	-
Chlorine	Acute	0.006	0.63	−0.04	**0.52**	**0.009**	**0.26**	**1.8**	**9.9E-06**	**0.94**
Chlorpyrifos	Acute	−0.04	0.64	−0.01	0.26	0.83	−0.01	-	-	-
Chlorpyrifos	Chronic	0.034	0.45	−0.01	−0.19	0.55	−0.02	-	-	-
Copper	Acute	0.027	0.87	−0.02	0.44	0.28	0.004	-	-	-
Copper	Chronic	0.012	0.11	0.061	−0.04	0.96	−0.05	-	-	-
DDT	Acute	0.1	0.53	−0.01	0.81	0.76	−0.01	-	-	-
Diazinon	Acute	−0.47	0.14	0.014	0.39	0.67	−0.01	-	-	-
Diazinon	Chronic	−***2.2***	***1E-03***	***0.47***	−4.7	0.55	−0.05	-	-	-
Dieldrin	Acute	0.01	0.17	0.016	−0.07	0.1	0.03	-	-	-
Endosulfan	Acute	0.015	0.69	−0.01	−0.51	0.46	−0.01	-	-	-
Endosulfan	Chronic	1.3E-05	0.99	−0.09	9E-04	0.95	−0.12	-	-	-
Endrin	Acute	0.009	0.55	−0.01	−0.04	0.66	−0.01	-	-	-
Glyphosate	Acute	−9.6	0.84	−0.03	11	0.8	−0.05	-	-	-
Glyphosate	Chronic	−0.04	0.85	−0.05	−32	0.15	0.13	-	-	-
Guthion	Acute	−0.03	0.47	−0.01	0.81	0.29	0.003	0.004	0.59	−0.07
Lindane	Acute	0.55	0.1	0.018	1.3	0.54	−0.01	-	-	-
Lindane	Chronic	0.003	0.74	−0.12	5.6	0.065	0.37	−0.01	0.91	−0.05
Malathion	Acute	**0.91**	**0.011**	**0.04**	0.59	0.91	−0.01	-	-	-
Malathion	Chronic	−0.21	0.68	−0.04	−2.3	0.097	0.2	-	-	-
Mercury	Acute	−0.05	0.49	−0.03	0.13	0.76	−0.08	−***1.4E-04***	***0.005***	***1***
Nickel	Acute	0.81	0.45	−0.02	−81	0.19	0.067	-	-	-
Nitrophenol	Acute	0.1	0.81	−0.08	−2.7	0.78	−0.1	0.008	0.41	−0.03
Parathion	Acute	0.16	0.18	0.017	−0.33	0.21	0.016	-	-	-
PCP	Acute	−***0.79***	***0.046***	***0.034***	3.6	0.47	−0.01	-	-	-
PCP	Chronic	−0	0.79	−0.05	0.17	0.81	−0.04	-	-	-
Phenol	Acute	−***9.3***	***0.014***	***0.079***	−5.9	0.95	−0.02	-	-	-
Phenol	Chronic	0.71	0.28	0.043	−16	0.26	0.13	-	-	-
TBTO	Acute	−4	0.39	−0.01	−25	0.78	−0.04	-	-	-
Toluene	Acute	14	0.2	0.034	51	0.87	−0.07	-	-	-
Zinc	Acute	0.52	0.28	0.005	0.002	1	−0.03	-	-	-
Zinc	Chronic	−0.0035	0.78	−0.1	−***0.2***	***0.012***	***0.79***	0.002	0.16	0.55

*A-R*^*2*^ Adjusted R^2^, *DDT* 1,1′-(2,2,2-Trichloroethane-1,1-diyl)bis(4-chlorobenzene), *PCP* Pentachlorophenol, *TBTO* Tributyltin oxide

Values in boldface indicate a significant (*p* ≤ 0.05) positive relationship

Values in *boldface italics* indicate a significant (*p* ≤ 0.05) negative relationship

## Discussion

Our results indicate that strong phylogenetic signal is rare in toxicity data from aquatic animals and that its frequency does not appear to be biased by organism life stage, exposure type, chemical origin, class or mode of action. We also found evidence of significant effects of experimental temperature, pH and hardness on chemical tolerance in a few datasets, although there were no consistent generalizable trends in how any of these variables affected toxicity across all chemicals considered. High variation in phylogenetic signal magnitude across different chemicals is consistent with the results of the similarly-sized analysis by Hylton et al. (2018), which found strong signal in just 10 of the 42 toxicity datasets they examined. In general, most of the previous studies (Buchwalter et al. 2008; Guénard et al. 2011; Hammond et al. 2012; Chiari et al. 2015; Hylton et al. 2018) that identified strong phylogenetic signal appear to have less taxonomic breadth in their data than what we used in our work, suggesting that phylogenetic signal might manifest more strongly at lower taxonomic levels (Carew et al. 2011). The absence of strong phylogenetic signal among the synthetic chemicals is particularly notable given that most are selective pesticides that were specifically developed to target insects. Instead, non-target organisms, including chordates, appear to be equally as sensitive as insects to many of these chemicals (see Fig. 3 for an example) which points to how pesticides can uniformly threaten all members of aquatic communities.

There are several factors that might explain the low frequency of strong phylogenetic signal in our results. First, it is possible that the sample sizes of some datasets, like chronic phenol (*n* = 10 species) and chronic zinc (*n* = 13 species), were simply too small to provide adequate statistical power to estimate λ. Sample sizes might increase if data quality collection parameters were less restrictive, however, this solution is not ideal because of the underlying variability in our toxicity datasets. This variation arises because an organism’s sensitivity to a toxicant may be affected by any number of environmental (temperature, pH, salinity, etc.), biological (size, sex, life stage, etc.) or chemical (mode of action, structure, solubility, etc.) factors. We aimed to reduce such variation and its effects by setting precise requirements for certain variables (CAS number, exposure media, test duration) during data collection and controlling others (life stage, temperature, pH and hardness) in the analyses. However, because of the sporadic availability of information on toxicity testing conditions in ECOTOX, we left variables such as chemical purity, dissolved oxygen content and test organism sex unrestricted to ensure there were enough data to perform the planned analyses. As such, we expect that some underlying variation remains in our data and may have obscured the phylogenetic signal in some instances.

Additionally, there is likely substantial statistical noise in the chronic toxicity data that does not affect acute data because of inconsistencies in the NOEC data we collected. Generated from *post hoc* tests after performing an ANOVA, the NOEC represents the highest concentration of a chemical that does not induce a response that differs significantly from the control. The biological effect used to determine a NOEC can vary widely between toxicity tests, and such experimental variation was present in the chronic toxicity datasets we collected (Table S2). For example, the chronic lindane dataset (*n* = 33 toxicity values) contained toxicity data that were measured using twelve different endpoints, which included but are not limited to mortality, behavior, population growth, reproduction, enzyme activity and hormone levels. Across all of the chronic datasets, each of the various effects had a very small average sample size of species ($\bar{x}$ < 4), the smallest unit utilized in our phylogenetic analysis, meaning that utilizing only NOECs derived from the same biological effect was not a viable approach in our study. Additionally, the NOEC is generally considered to be a relatively poor indicator of safe chemical concentrations (Crane and Newman 2000), and as a result there has been a strong push in ecotoxicology to utilize alternative measures of chronic toxicity (Warne and van Dam 2008; Laskowski 1995; Kooijman 1996). Given this criticism and substantial issue of experimental variation, NOEC data does not appear suitable for use in relatedness-based cross-species extrapolation. Opportunities to work with alternatives, however, are minimal given that chronic data are severely limited or even nonexistent for many chemicals (de Zwart 2001). The paradox created by this shortage and the requirement of existing data for typical CSE approaches means we are unlikely to be able to comprehensively fill gaps in the chronic toxicity database using these methods. Instead, statistical approaches that extrapolate chronic values using acute data (Duboudin et al. 2004; Hiki and Iwasaki 2020) appear better suited to this challenge. Given that the endpoints and test protocols used to measure acute toxicity are more uniform, it is more likely that phylogenetic methods can be used to address the gaps in the acute database.

The value of phylogenetic approaches to CSE arises from the concept that species data cannot be considered independent observations (Felsenstein 1985). All species are related within a hierarchical phylogenetic tree, so similar phenotype values among species could be a product of limited divergence from a shared common ancestor or convergent evolution (Stone et al. 2011). Most standard statistical tests assume datapoints to be independent, meaning that using such methods to analyze a dataset heavily influenced by evolutionary history could lead to spurious results. Phylogenetically-informed methods avoid this issue by explicitly accounting for possible phylogenetic structure in a dataset. In this context, the best-studied method of phylogenetic CSE is the phylogenetic eigenvector map (PEM; Guénard, Legendre, and Peres-Neto 2013; Guénard et al. 2014). A PEM is a set of eigenfunctions derived from a phylogeny that describe the magnitude of various possible phylogenetic patterns (i.e. phylogenetic signal values) in a dataset. A subset of these eigenfunctions are then utilized as the independent variables in a regression model that generates estimates of trait values for species on the phylogeny that lack data. PEMs have also been combined with descriptors of chemical properties in a bilinear modelling approach that can predict the toxicity of multiple chemicals to many species (Guénard et al. 2014), which represents a substantial expansion of conventional modelling efforts.

Approaching toxicity data with an evolutionary perspective may benefit pollution management efforts beyond cross-species extrapolation. Here, we identified patterns of sensitivity in the datasets that exhibited strong phylogenetic signal that correspond with descriptions from the literature of how these chemicals affect different taxa. For example, neurotoxins like guthion, an organophosphate insecticide, are typically considered more toxic to invertebrates than vertebrates (Legradi et al. 2018), which is reflected in our results (Fig. 2). Similarly, freshwater mussels have been found to be more tolerant of chlorine than other aquatic species (Valenti et al. 2006) which matches with our evaluation of the molluscs in our acute toxicity dataset for chlorine (Fig. 1). These similarities suggest that phylogenetic assessments of toxicity data can provide reliable insights into patterns of sensitivity which, in a regulatory context, could aid in water quality criteria development. For instance, by referring to our guthion plot (Fig. 2), it becomes clear that amphipods are some of the most acutely sensitive taxa to guthion. A toxicity dataset for guthion water quality criteria could then be specifically assembled to include amphipods rather than relying on the EPA’s taxonomic requirements (Stephan et al. 1985) to ensure their representation. While setting unique taxonomic requirements for every chemical is unrealistic, knowledge of patterns of sensitivity could be used to complement other efforts to modernize water quality criteria.

## Conclusions

Given that understanding of the concentrations of chemicals that adversely affect species is a vital component of the risk assessment process, it is critical that we address the major gaps in our toxicity database. Phylogenetic methods of cross-species extrapolation represent a possible alternative to laboratory testing, provided that chemical sensitivity is strongly influenced by evolutionary relationships. We found that strong phylogenetic signal is apparent at high taxonomic levels for only a small subset of chemicals in both acute and chronic data, which was surprising given the specific targets of many pesticides included in this study. For those acute toxicity datasets that do feature strong signal, phylogenetic tools provide a framework with which we can reliably assess patterns in chemical sensitivity and a means of avoiding the statistical pitfalls associated with phylogenetically structured data. Moving forwards, we recommend that future efforts in this field evaluate the phylogenetic signal in their data as a preliminary analytical step to ensure the selection of the appropriate method of cross-species extrapolation.

## Supplementary Information

Supplementary Information

## Data Availability

Data pertaining to this manuscript are available on Zenodo at 10.5281/zenodo.8222812.
