# Supplementary material for: Phylogeny predicts sensitivity in aquatic animals for only a minority of chemicals
Source: Ecotoxicology. 2024 Jul 22;33(8):921–36. doi: 10.1007/s10646-024-02791-7 (PMC11399186; doi:10.1007/s10646-024-02791-7)
Supplement: Supplementary file 1 — Supplementary Information [file 10646_2024_2791_MOESM1_ESM.docx]

**Table S1** Toxicity dataset sample sizes before and after filtering for availability of experimental life stage, temperature, pH and hardness information

| **Chemical** | **Exposure** | **Dataset Variation** | | | | | | | | | |
| --- | --- | --- | --- | --- | --- | --- | --- | --- | --- | --- | --- |
|  |  | **Complete** | | **Subadult** | | **Temperature** | | **pH** | | **Hardness** | |
|  |  | **Tox  Values** | **Species** | **Tox  Values** | **Species** | **Tox  Values** | **Species** | **Tox  Values** | **Species** | **Tox  Values** | **Species** |
| Ammonia | Acute | 95 | 50 | 43 | 19 | 76 | 41 | 74 | 39 | - | - |
| Atrazine | Acute | 144 | 60 | 59 | 30 | 124 | 57 | 88 | 44 | - | - |
| Atrazine | Chronic | 742 | 71 | 258 | 41 | 673 | 66 | 492 | 45 | - | - |
| Cadmium | Acute | 233 | 80 | 111 | 31 | 183 | 62 | 126 | 45 | 68 | 23 |
| Cadmium | Chronic | 164 | 14 | 12 | 5 | 150 | 11 | 104 | 5 | 99 | 3 |
| Chlorine | Acute | 116 | 28 | 24 | 6 | 102 | 23 | 100 | 22 | - | - |
| Chlorpyrifos | Acute | 993 | 112 | 129 | 56 | 821 | 100 | 194 | 77 | - | - |
| Chlorpyrifos | Chronic | 512 | 57 | 172 | 30 | 390 | 41 | 281 | 30 | - | - |
| Copper | Acute | 435 | 100 | 176 | 37 | 288 | 64 | 306 | 52 | 238 | 37 |
| Copper | Chronic | 523 | 34 | 185 | 19 | 341 | 28 | 296 | 24 | 189 | 12 |
| DDT | Acute | 413 | 115 | 78 | 37 | 381 | 101 | 224 | 77 | - | - |
| Diazinon | Acute | 274 | 91 | 95 | 47 | 216 | 82 | 144 | 61 | - | - |
| Diazinon | Chronic | 198 | 21 | 51 | 15 | 139 | 18 | 121 | 14 | - | - |
| Dieldrin | Acute | 280 | 69 | 69 | 27 | 247 | 61 | 140 | 58 | - | - |
| Endosulfan | Acute | 489 | 109 | 110 | 47 | 333 | 88 | 248 | 73 | - | - |
| Endosulfan | Chronic | 56 | 16 | 50 | 12 | 25 | 13 | 21 | 10 | - | - |
| Endrin | Acute | 227 | 76 | 58 | 32 | 214 | 70 | 152 | 60 | - | - |
| Glyphosate | Acute | 115 | 35 | 41 | 17 | 99 | 30 | 65 | 20 | - | - |
| Glyphosate | Chronic | 238 | 26 | 130 | 19 | 207 | 22 | 97 | 11 | - | - |
| Guthion | Acute | 274 | 55 | 54 | 23 | 242 | 53 | 197 | 49 | - | - |
| Lindane | Acute | 253 | 102 | 56 | 32 | 218 | 94 | 188 | 85 | - | - |
| Lindane | Chronic | 33 | 13 | 7 | 6 | 19 | 9 | 18 | 8 | - | - |
| Malathion | Acute | 422 | 148 | 127 | 68 | 368 | 138 | 294 | 114 | - | - |
| Malathion | Chronic | 223 | 21 | 85 | 13 | 201 | 21 | 180 | 11 | - | - |
| Mercury | Acute | 75 | 32 | 37 | 10 | 57 | 22 | 33 | 13 | 11 | 11 |
| Nickel | Acute | 41 | 20 | 16 | 5 | 28 | 18 | 33 | 14 | 9 | 9 |
| Nitrophenol | Acute | 52 | 16 | 21 | 6 | 45 | 15 | 28 | 12 | - | - |
| Parathion | Acute | 184 | 54 | 66 | 27 | 159 | 52 | 108 | 38 | - | - |
| PCP | Acute | 490 | 116 | 172 | 45 | 382 | 89 | 356 | 84 | - | - |
| PCP | Chronic | 121 | 27 | 72 | 20 | 95 | 21 | 80 | 24 | - | - |
| Phenol | Acute | 262 | 79 | 78 | 21 | 205 | 64 | 179 | 49 | - | - |
| Phenol | Chronic | 47 | 10 | 22 | 7 | 32 | 9 | 26 | 6 | - | - |
| TBTO | Acute | 103 | 47 | 25 | 15 | 64 | 33 | 52 | 28 | - | - |
| Toluene | Acute | 85 | 28 | 40 | 12 | 74 | 22 | 61 | 16 | - | - |
| Zinc | Acute | 149 | 62 | 64 | 20 | 111 | 43 | 86 | 33 | 24 | 24 |
| Zinc | Chronic | 77 | 14 | 14 | 5 | 64 | 11 | 24 | 6 | 8 | 4 |

Abbreviations: DDT = 1,1′-(2,2,2-Trichloroethane-1,1-diyl)bis(4-chlorobenzene), PCP = Pentachlorophenol, TBTO = Tributyltin oxide

**Table S2** Summary of NOEC effects information in chronic toxicity datasets

| **Chemical** | **Effects** | **Species in  Dataset** | **Average Species  per Effect** |
| --- | --- | --- | --- |
| Ammonia | 7 | 9 | 1.29 |
| Atrazine | 20 | 71 | 3.55 |
| Cadmium | 10 | 14 | 1.4 |
| Chlorpyrifos | 19 | 60 | 3.16 |
| Copper | 16 | 34 | 2.12 |
| Diazinon | 11 | 21 | 1.91 |
| Dieldrin | 4 | 9 | 2.25 |
| Endosulfan | 8 | 16 | 2 |
| Glyphosate | 15 | 26 | 1.73 |
| Lindane | 12 | 13 | 1.08 |
| Malathion | 15 | 21 | 1.4 |
| PCP | 13 | 27 | 2.08 |
| Phenol | 5 | 10 | 2 |
| Zinc | 8 | 14 | 1.75 |


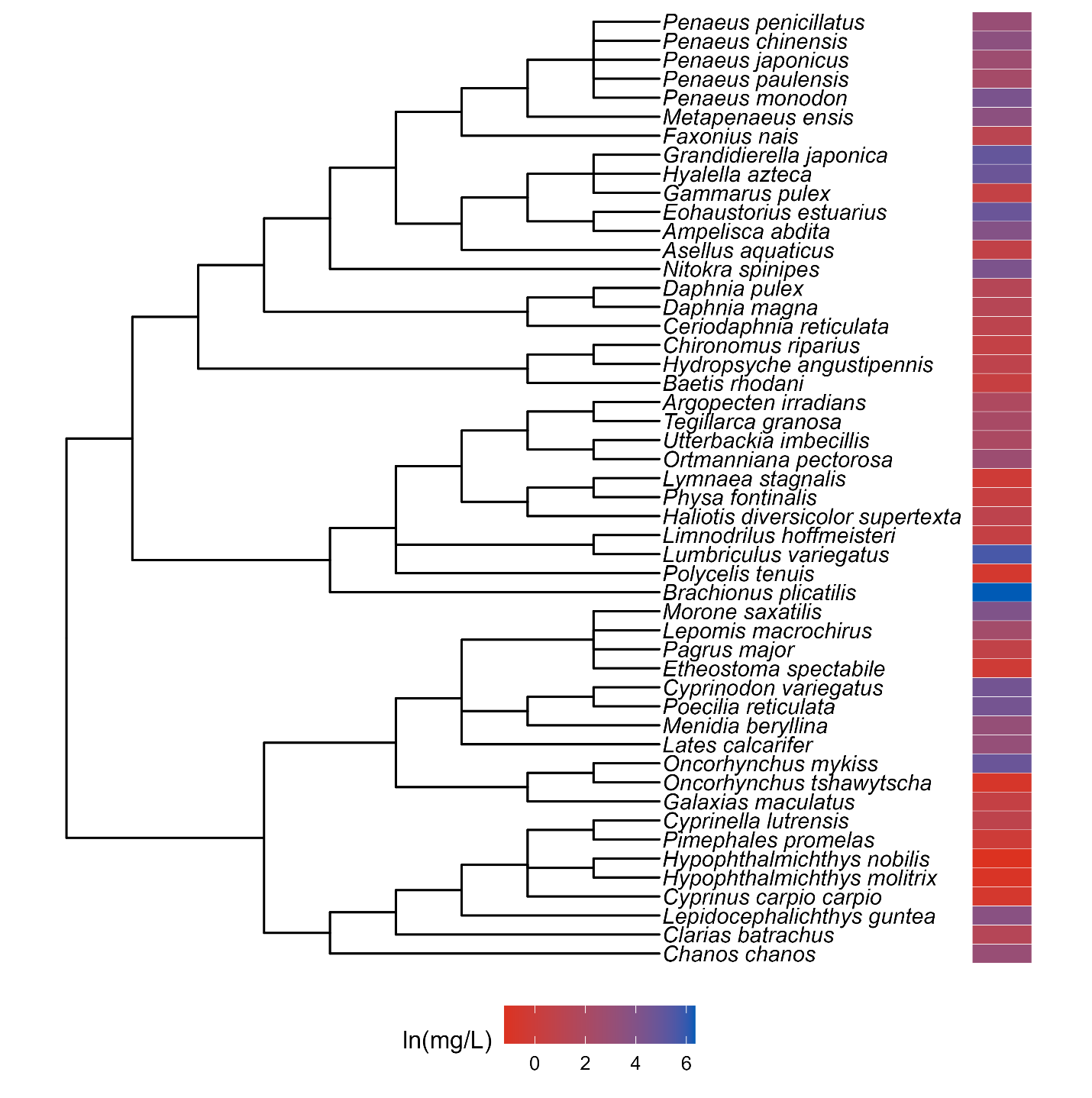


**Fig. S1** Phylogenetic tree and toxicity data heatmap for the complete acute ammonia dataset (λ = 7.3E-05). The colored bar next to each species represents its relative sensitivity to the chemical. A red bar indicates a high degree of sensitivity (i.e. small amount of chemical causes toxic effect), while a blue bar indicates low sensitivity (i.e. large amount of chemical causes toxic effect).


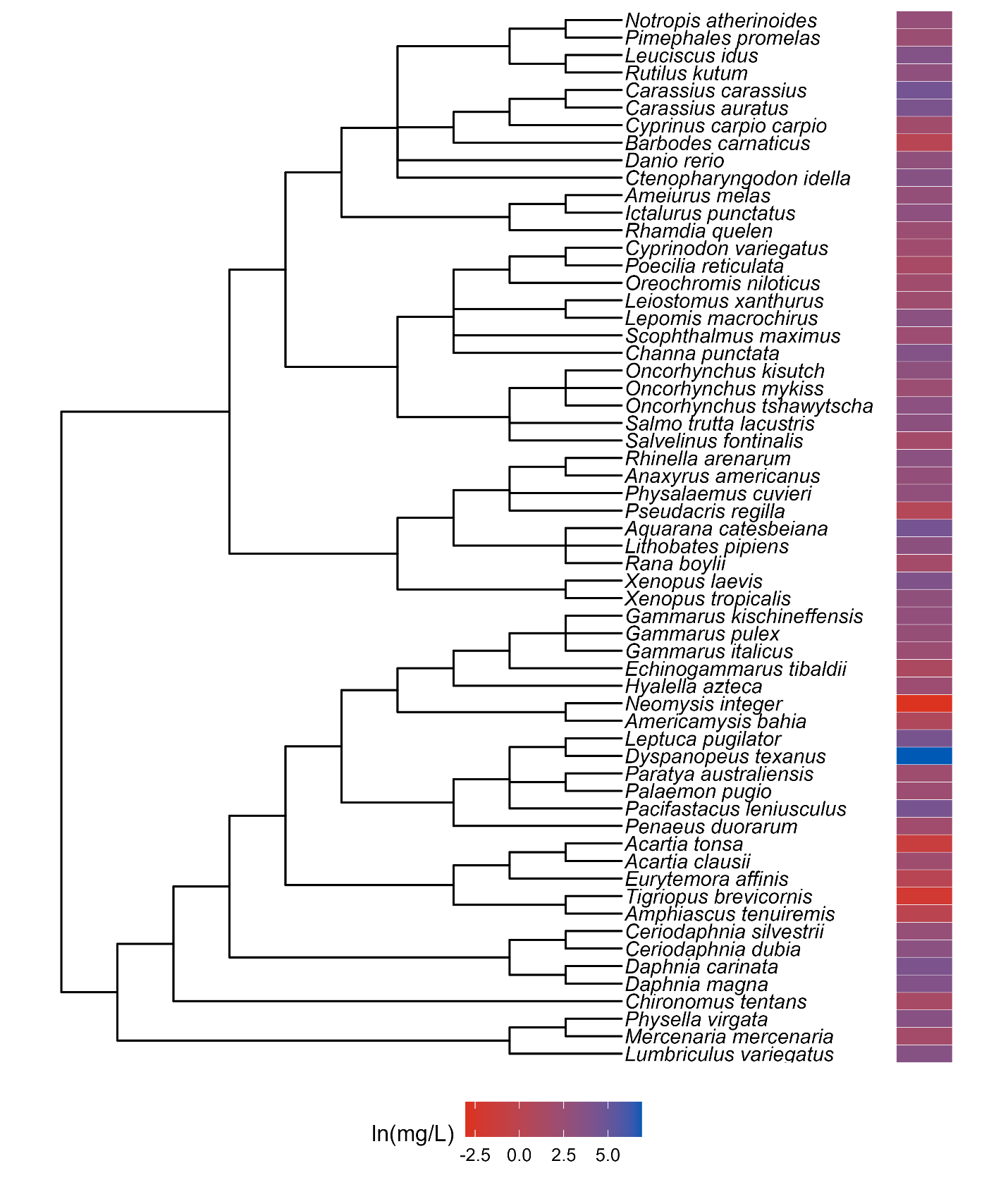


**Fig. S2** Phylogenetic tree and toxicity data heatmap for the complete acute atrazine dataset (λ = 7.3E-05). The colored bar next to each species represents its relative sensitivity to the chemical. A red bar indicates a high degree of sensitivity (i.e. small amount of chemical causes toxic effect), while a blue bar indicates low sensitivity (i.e. large amount of chemical causes toxic effect).


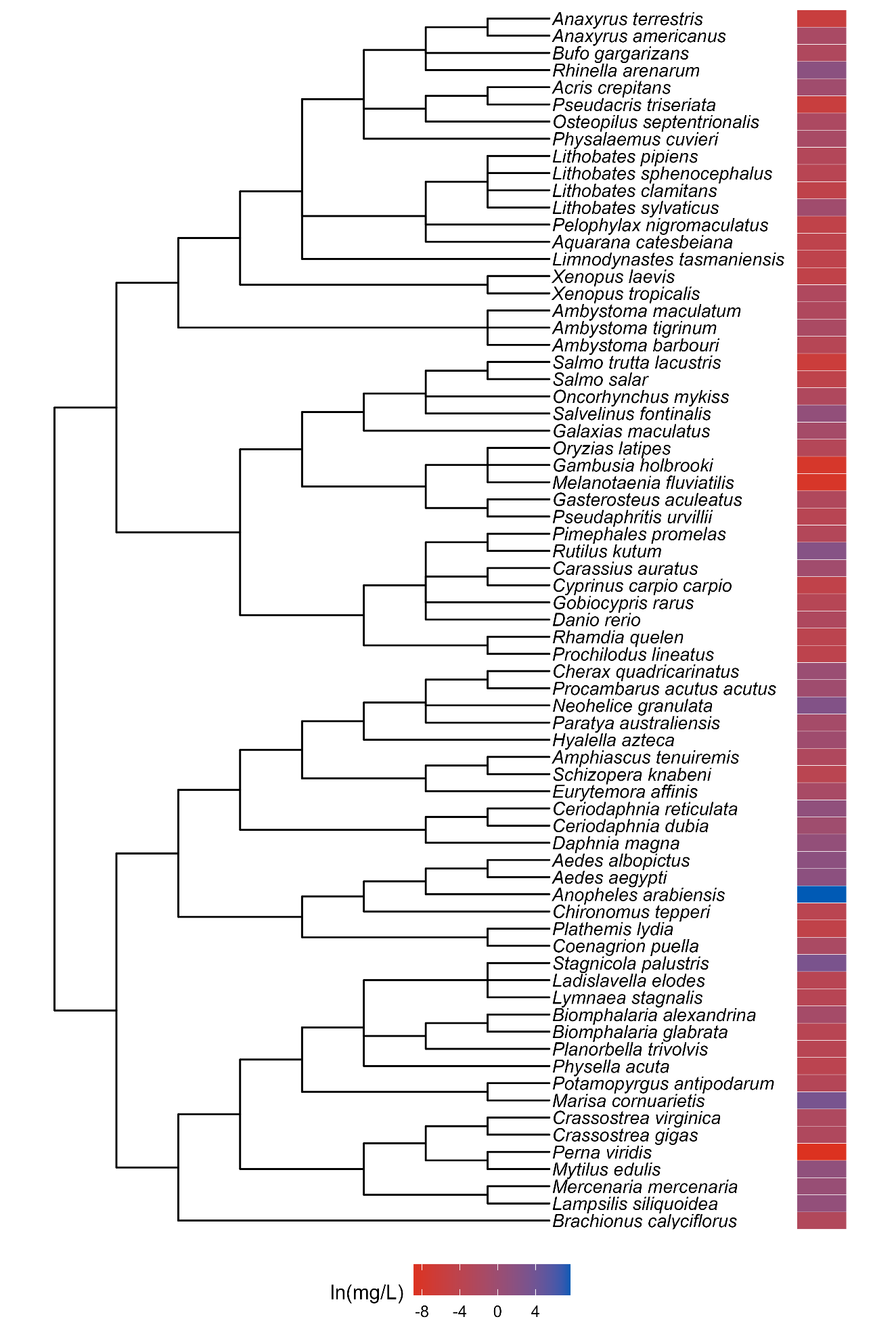


**Fig. S3** Phylogenetic tree and toxicity data heatmap for the complete chronic atrazine dataset (λ = 7.3E-05). The colored bar next to each species represents its relative sensitivity to the chemical. A red bar indicates a high degree of sensitivity (i.e. small amount of chemical causes toxic effect), while a blue bar indicates low sensitivity (i.e. large amount of chemical causes toxic effect).


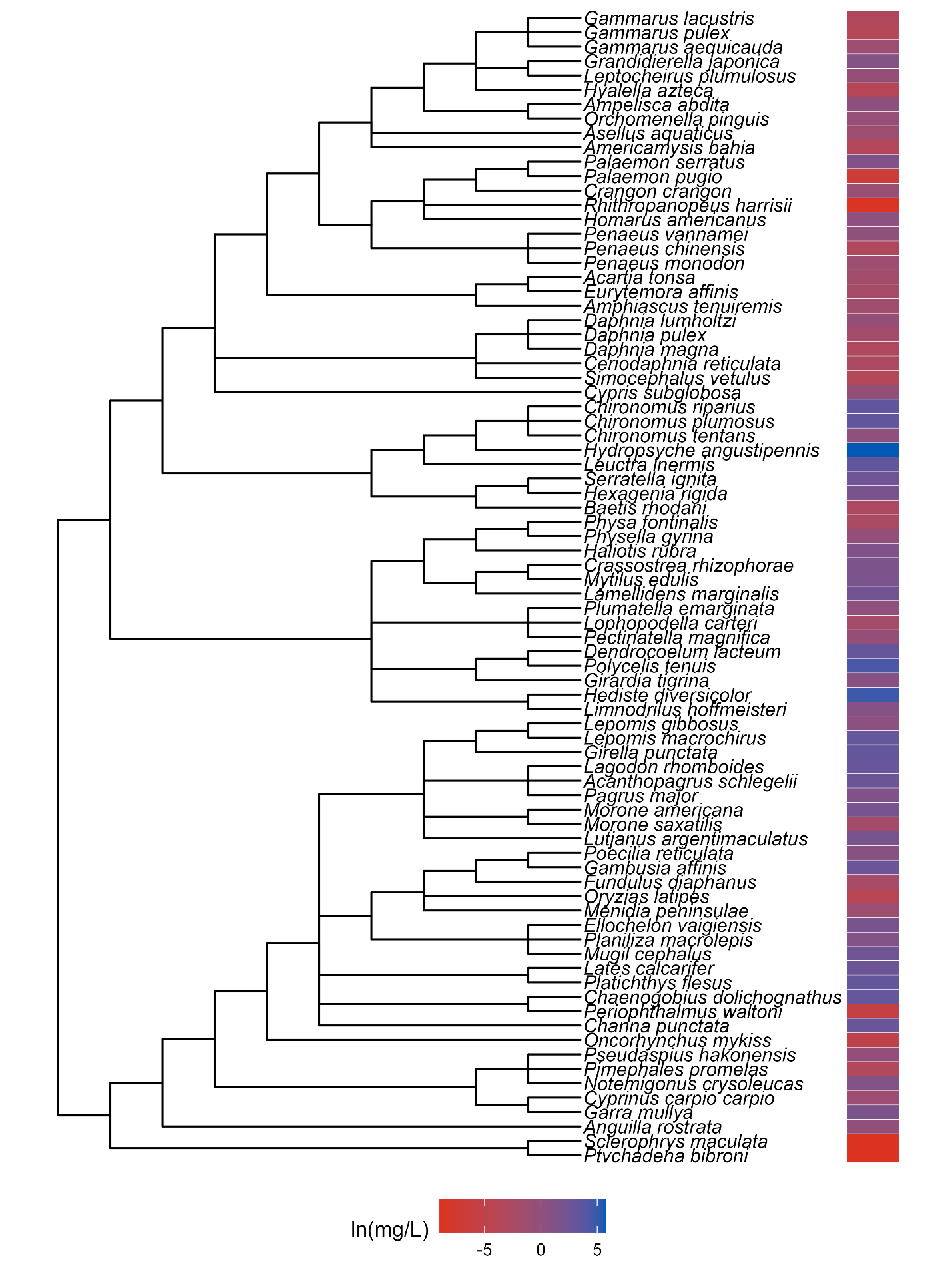


**Fig. S4** Phylogenetic tree and toxicity data heatmap for the complete acute cadmium dataset (λ = 7.3E-05). The colored bar next to each species represents its relative sensitivity to the chemical. A red bar indicates a high degree of sensitivity (i.e. small amount of chemical causes toxic effect), while a blue bar indicates low sensitivity (i.e. large amount of chemical causes toxic effect).


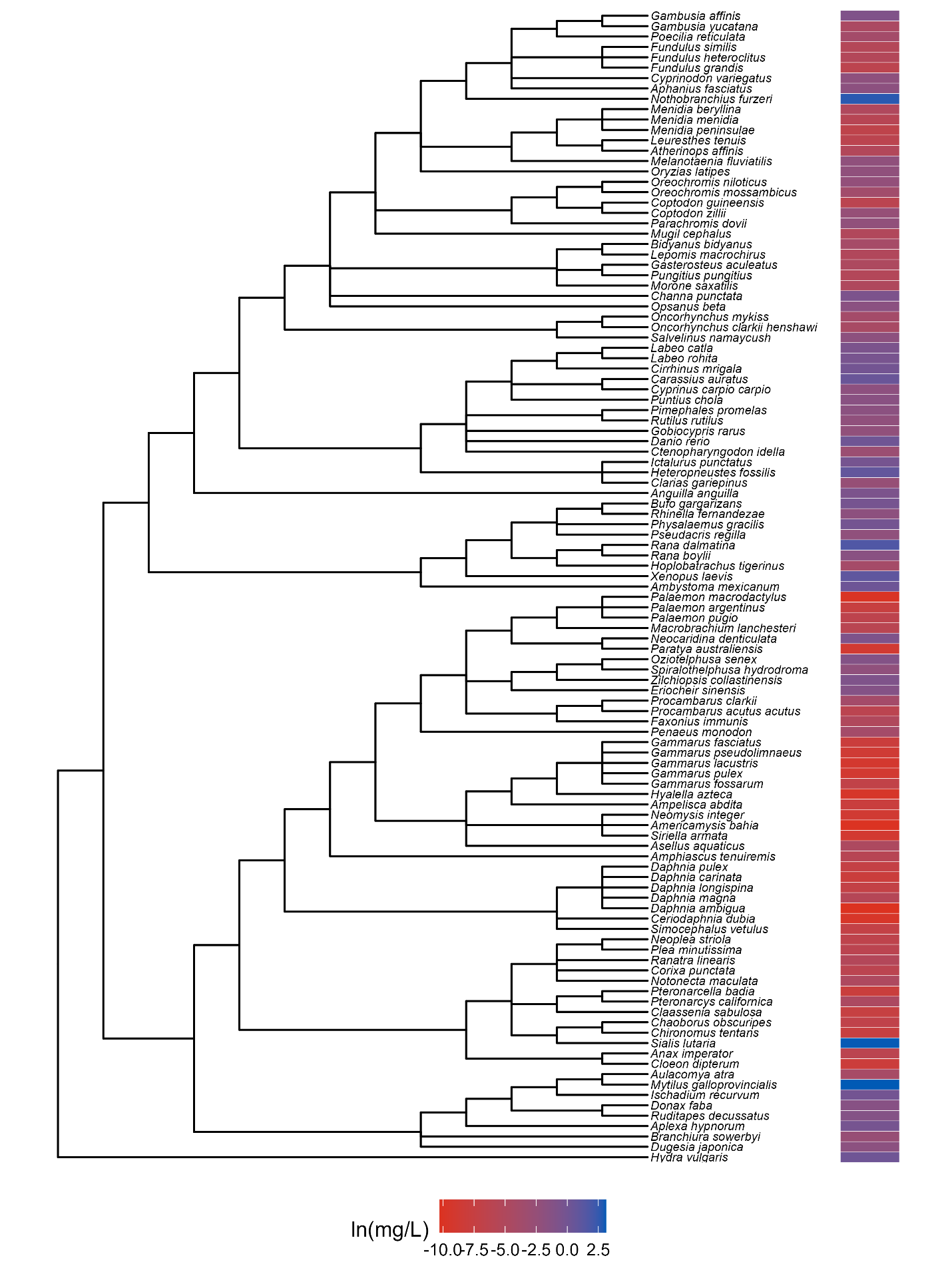


**Fig. S5** Phylogenetic tree and toxicity data heatmap for the complete acute chlorpyrifos dataset (λ = 7.3E-05). The colored bar next to each species represents its relative sensitivity to the chemical. A red bar indicates a high degree of sensitivity (i.e. small amount of chemical causes toxic effect), while a blue bar indicates low sensitivity (i.e. large amount of chemical causes toxic effect).


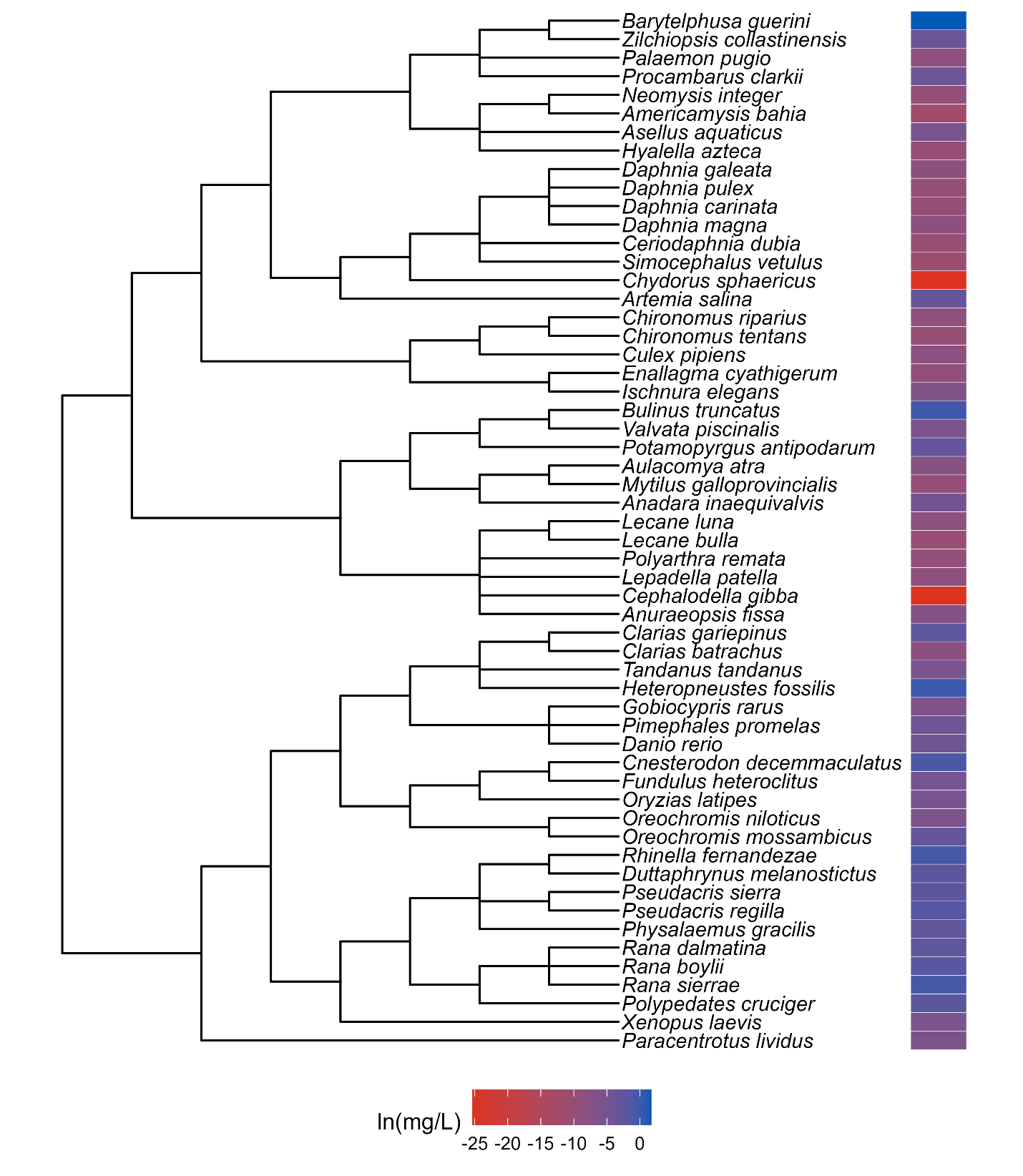


**Fig. S6** Phylogenetic tree and toxicity data heatmap for the complete chronic chlorpyrifos dataset (λ = 7.3E-05). The colored bar next to each species represents its relative sensitivity to the chemical. A red bar indicates a high degree of sensitivity (i.e. small amount of chemical causes toxic effect), while a blue bar indicates low sensitivity (i.e. large amount of chemical causes toxic effect).


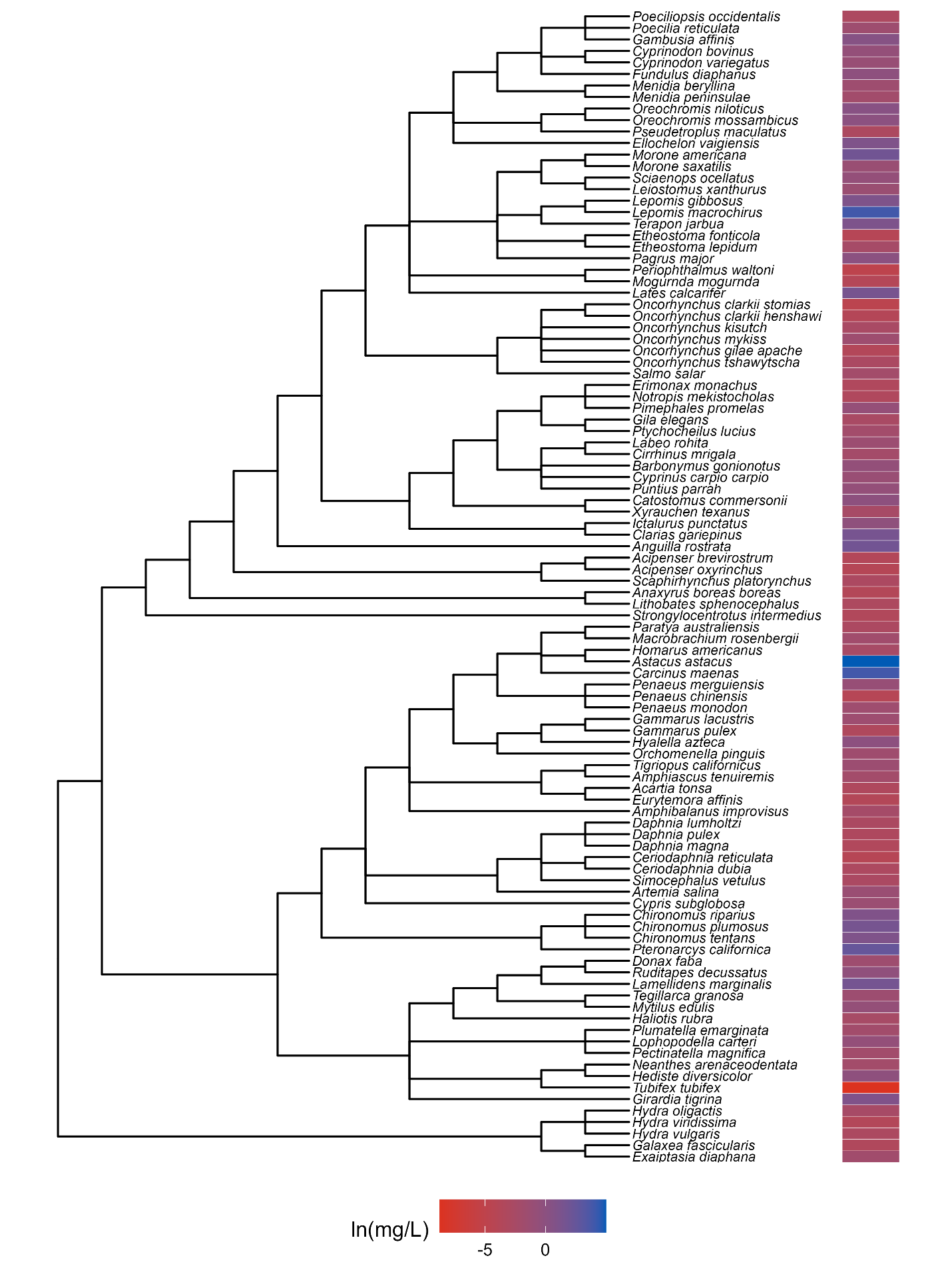


**Fig. S7** Phylogenetic tree and toxicity data heatmap for the complete acute copper dataset (λ = 7.3E-05). The colored bar next to each species represents its relative sensitivity to the chemical. A red bar indicates a high degree of sensitivity (i.e. small amount of chemical causes toxic effect), while a blue bar indicates low sensitivity (i.e. large amount of chemical causes toxic effect).


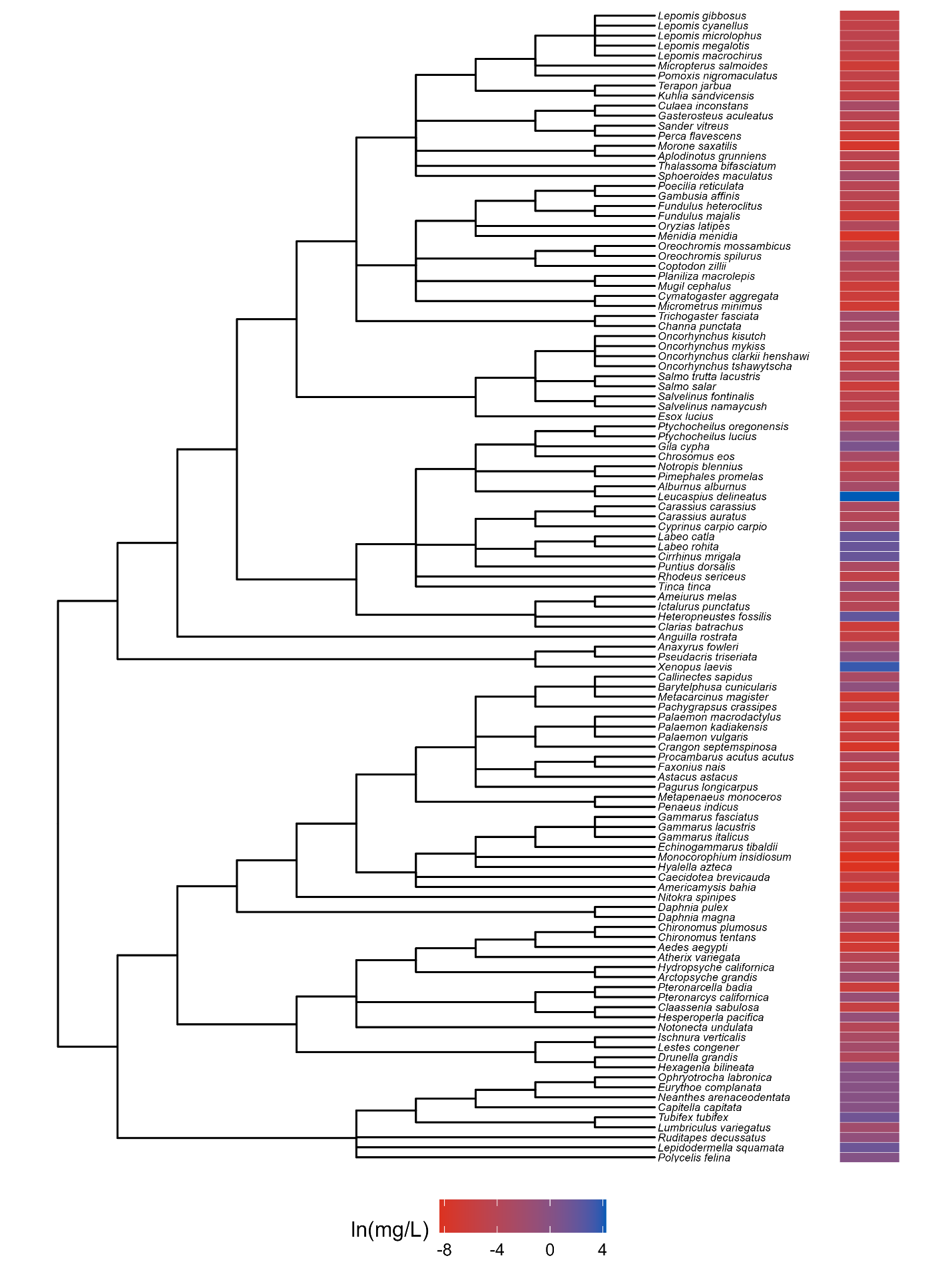


**Fig. S8** Phylogenetic tree and toxicity data heatmap for the complete acute DDT dataset (λ = 0.00065). The colored bar next to each species represents its relative sensitivity to the chemical. A red bar indicates a high degree of sensitivity (i.e. small amount of chemical causes toxic effect), while a blue bar indicates low sensitivity (i.e. large amount of chemical causes toxic effect). (DDT = 1,1′-(2,2,2-Trichloroethane-1,1-diyl)bis(4-chlorobenzene)


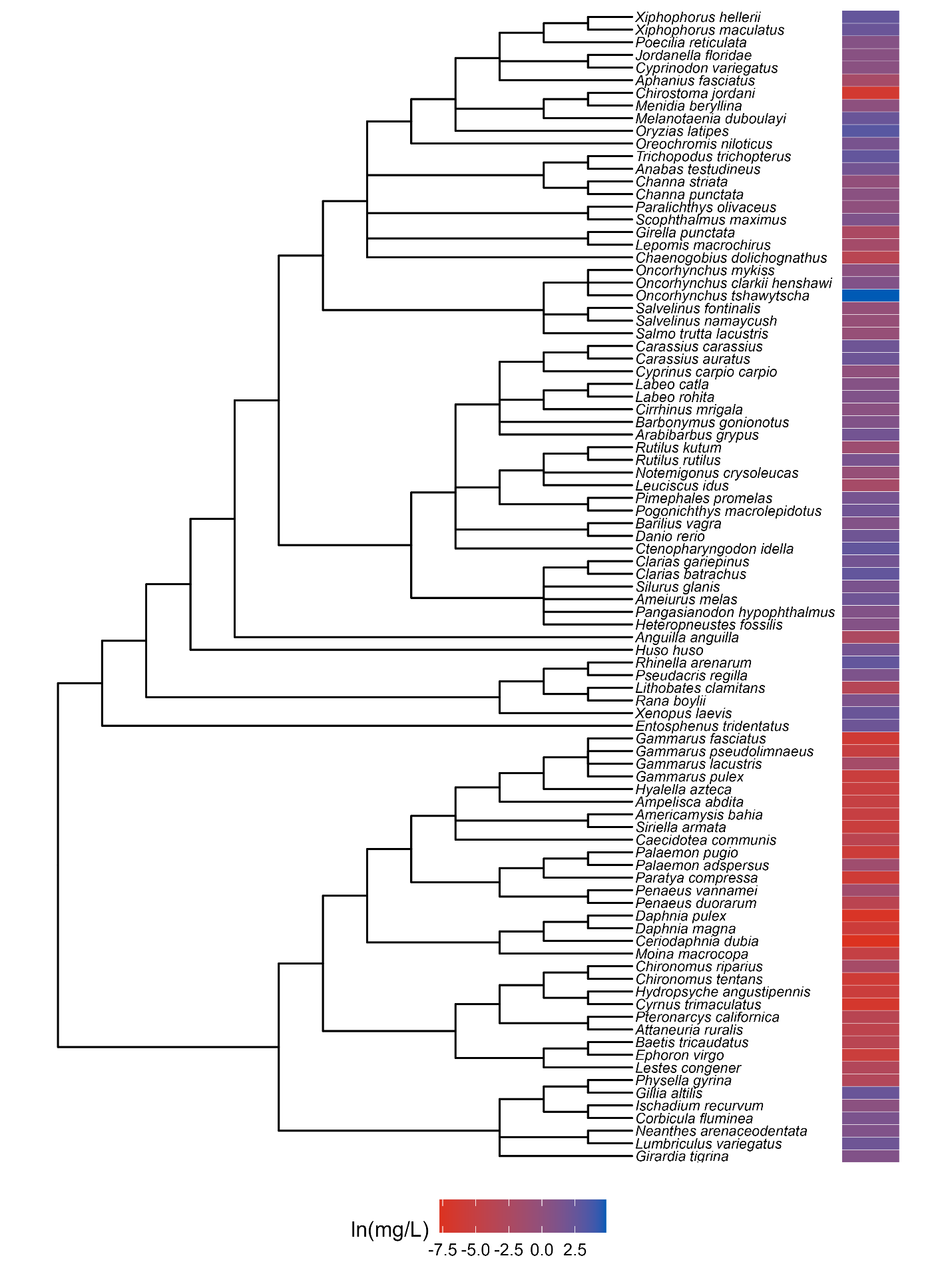


**Fig. S9** Phylogenetic tree and toxicity data heatmap for the complete acute diazinon dataset (λ = 0.023). The colored bar next to each species represents its relative sensitivity to the chemical. A red bar indicates a high degree of sensitivity (i.e. small amount of chemical causes toxic effect), while a blue bar indicates low sensitivity (i.e. large amount of chemical causes toxic effect).

**
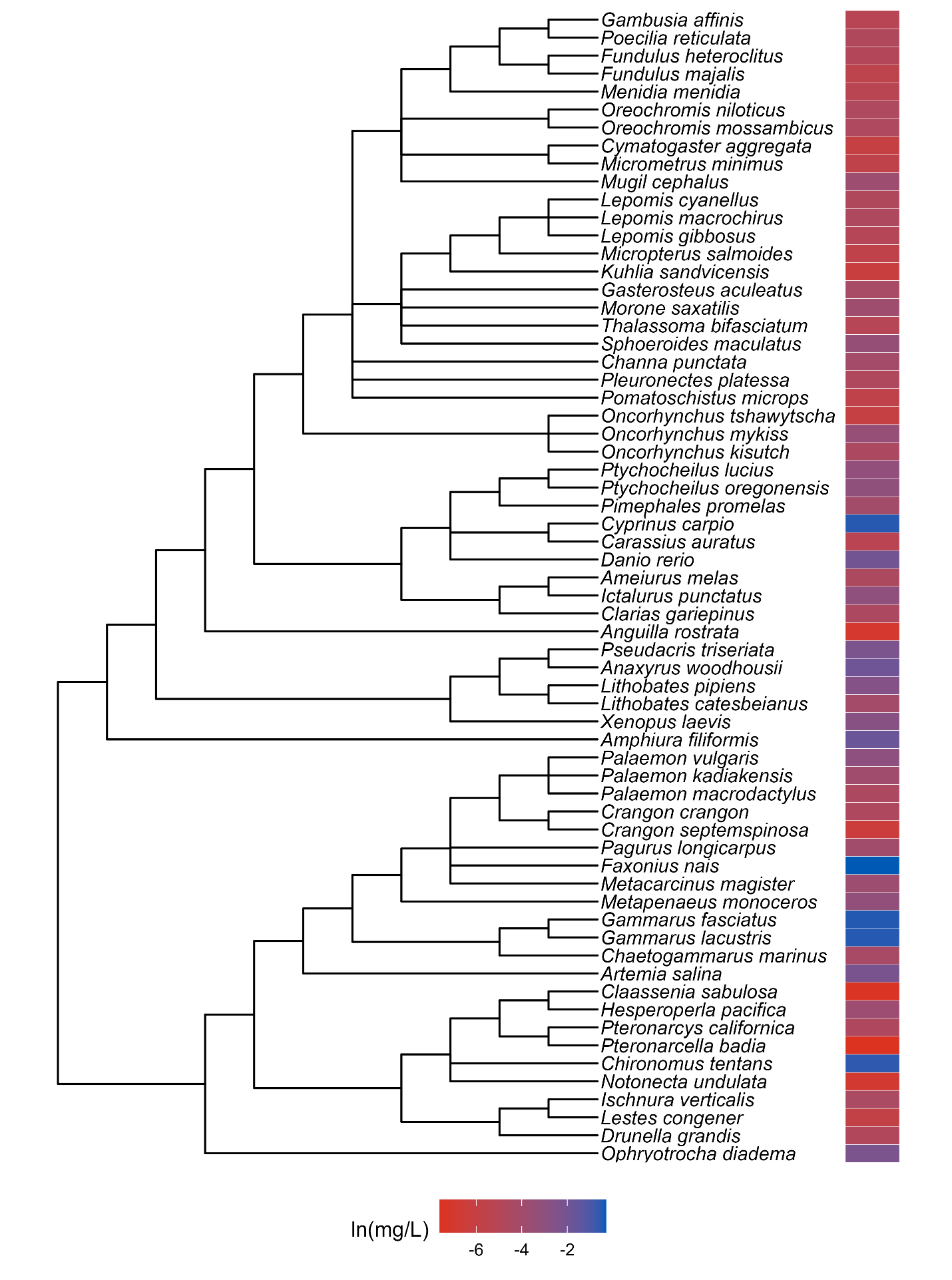
**

**Fig. S10** Phylogenetic tree and toxicity data heatmap for the complete acute dieldrin dataset (λ = 7.3E-05). The colored bar next to each species represents its relative sensitivity to the chemical. A red bar indicates a high degree of sensitivity (i.e. small amount of chemical causes toxic effect), while a blue bar indicates low sensitivity (i.e. large amount of chemical causes toxic effect).


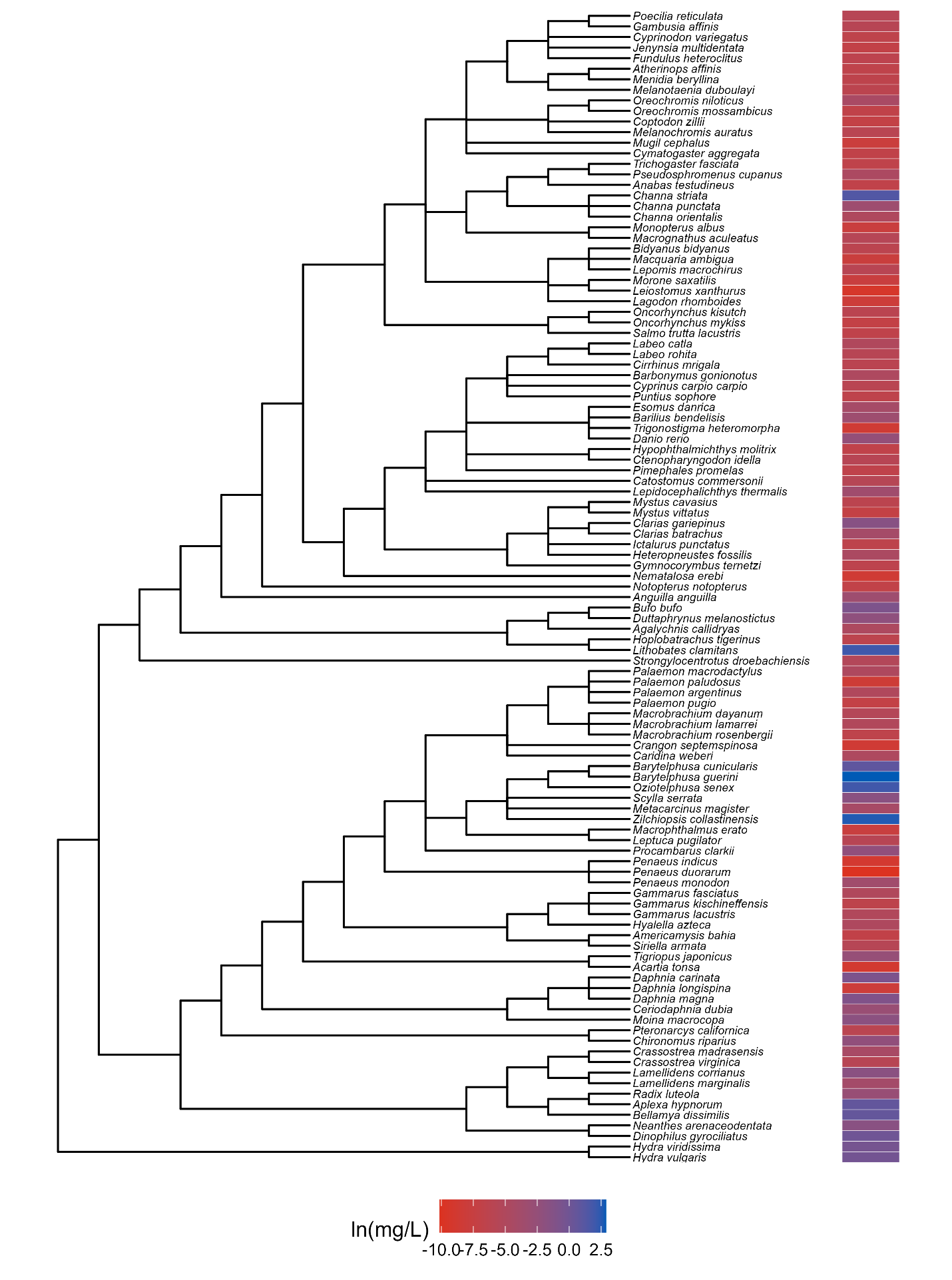


**Fig. S11** Phylogenetic tree and toxicity data heatmap for the complete acute endosulfan dataset (λ = 0.03). The colored bar next to each species represents its relative sensitivity to the chemical. A red bar indicates a high degree of sensitivity (i.e. small amount of chemical causes toxic effect), while a blue bar indicates low sensitivity (i.e. large amount of chemical causes toxic effect).


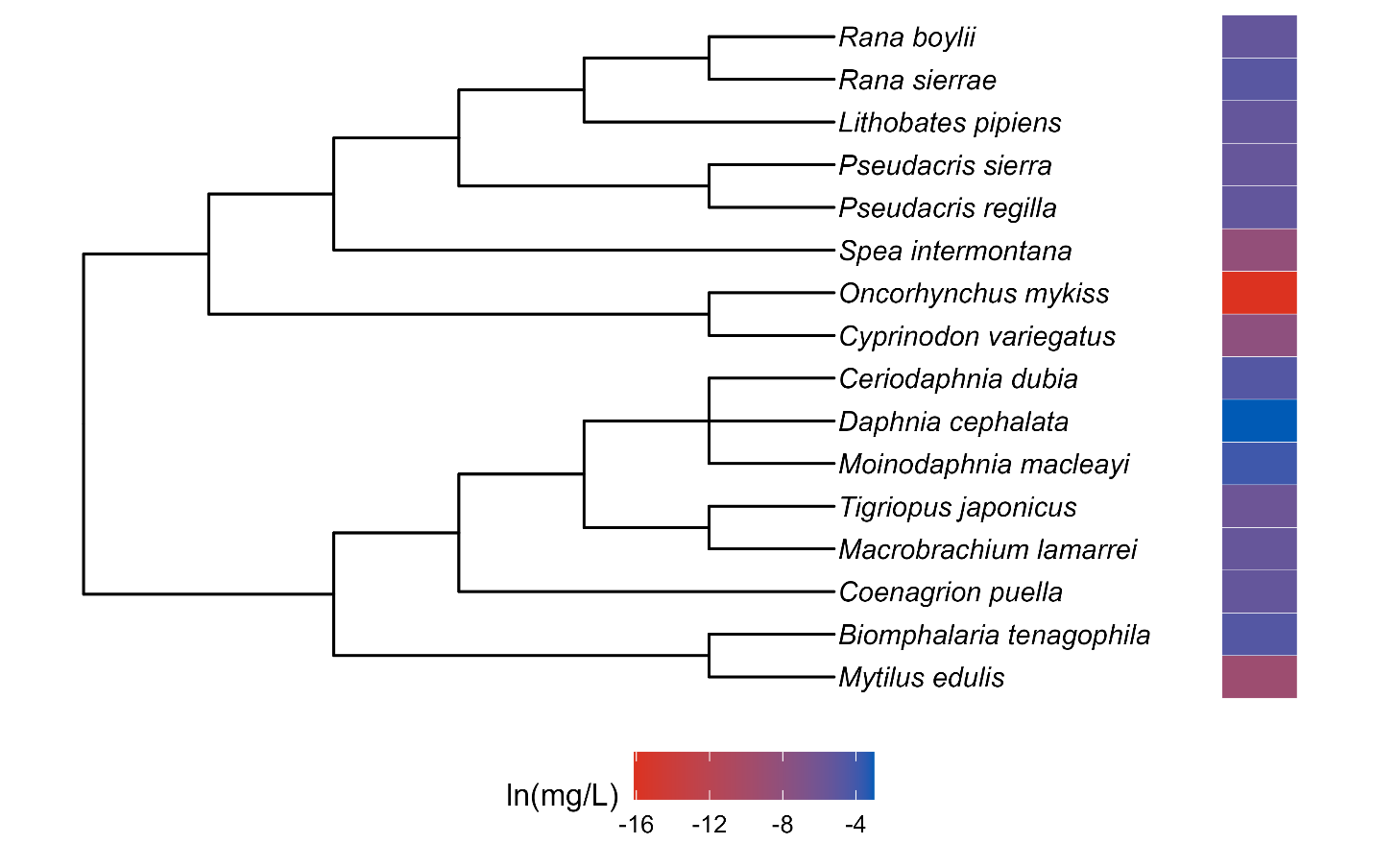


**Fig. S12** Phylogenetic tree and toxicity data heatmap for the complete chronic endosulfan dataset (λ = 0.11). The colored bar next to each species represents its relative sensitivity to the chemical. A red bar indicates a high degree of sensitivity (i.e. small amount of chemical causes toxic effect), while a blue bar indicates low sensitivity (i.e. large amount of chemical causes toxic effect).

**
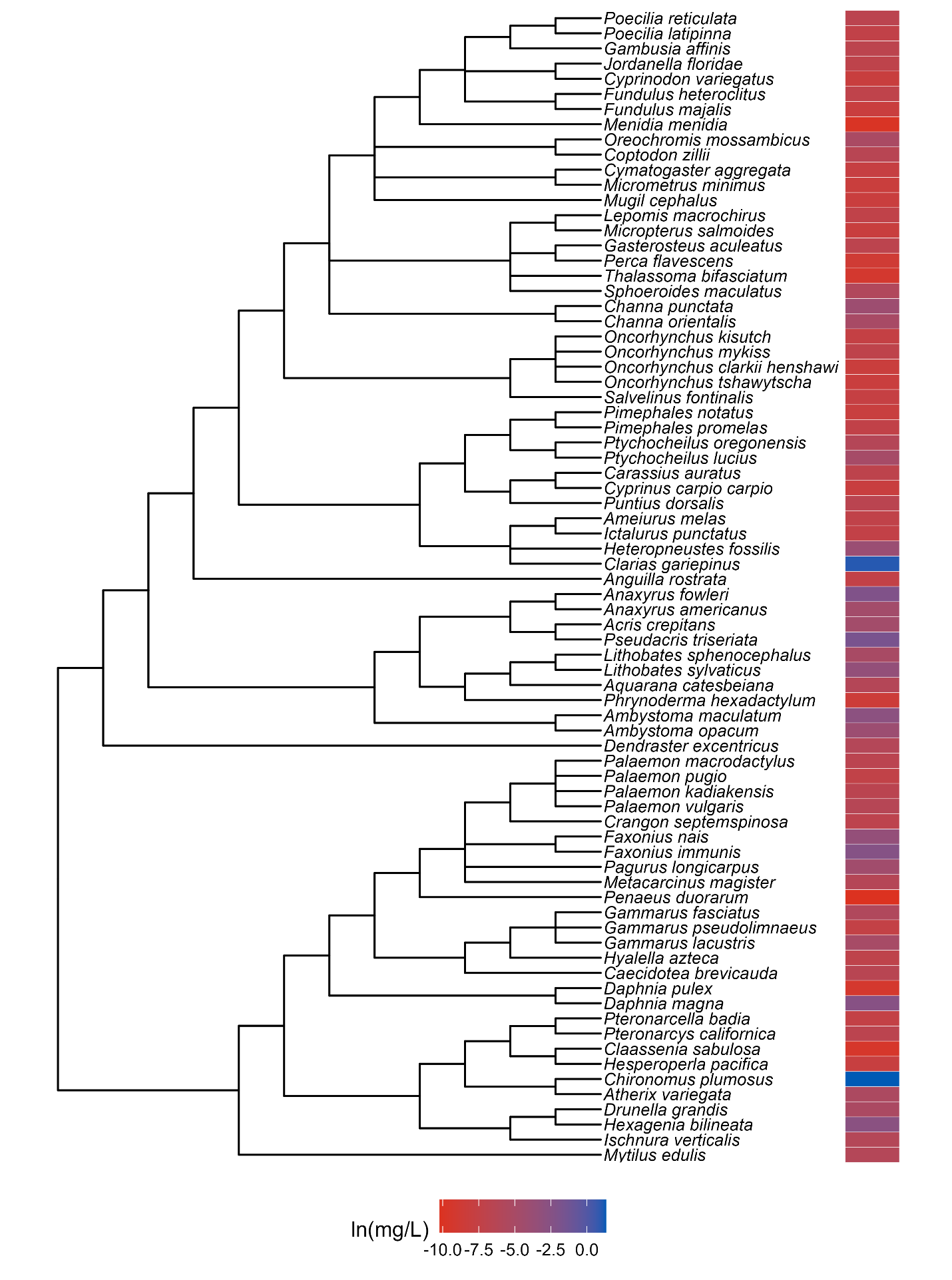
**

**Fig. S13** Phylogenetic tree and toxicity data heatmap for the complete acute endrin dataset (λ = 7.3E-05). The colored bar next to each species represents its relative sensitivity to the chemical. A red bar indicates a high degree of sensitivity (i.e. small amount of chemical causes toxic effect), while a blue bar indicates low sensitivity (i.e. large amount of chemical causes toxic effect).


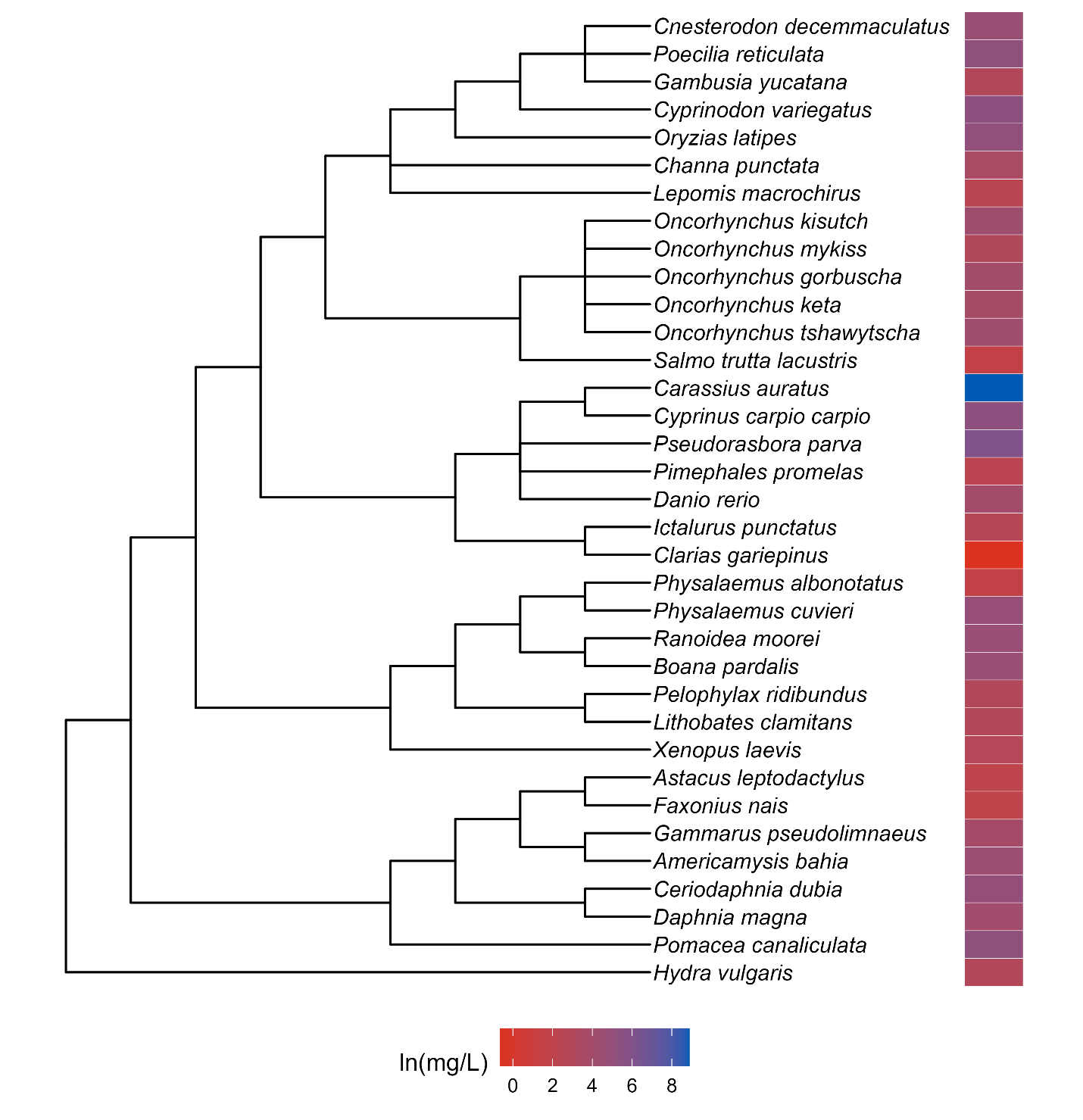


**Fig. S14** Phylogenetic tree and toxicity data heatmap for the complete acute glyphosate dataset (λ = 7.4E-05). The colored bar next to each species represents its relative sensitivity to the chemical. A red bar indicates a high degree of sensitivity (i.e. small amount of chemical causes toxic effect), while a blue bar indicates low sensitivity (i.e. large amount of chemical causes toxic effect).


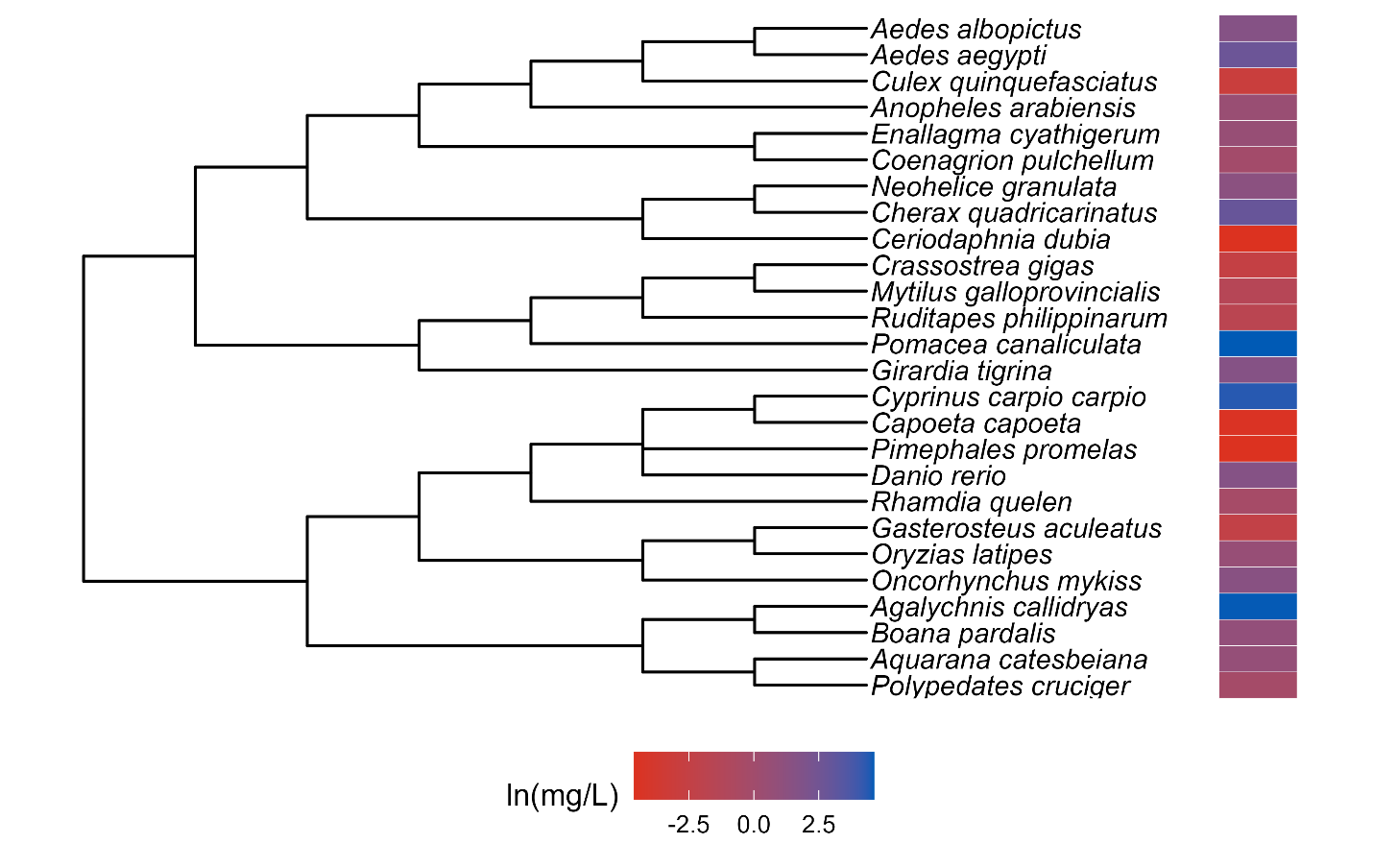


**Fig. S15** Phylogenetic tree and toxicity data heatmap for the complete chronic glyphosate dataset (λ = 7.3E-05). The colored bar next to each species represents its relative sensitivity to the chemical. A red bar indicates a high degree of sensitivity (i.e. small amount of chemical causes toxic effect), while a blue bar indicates low sensitivity (i.e. large amount of chemical causes toxic effect).

**
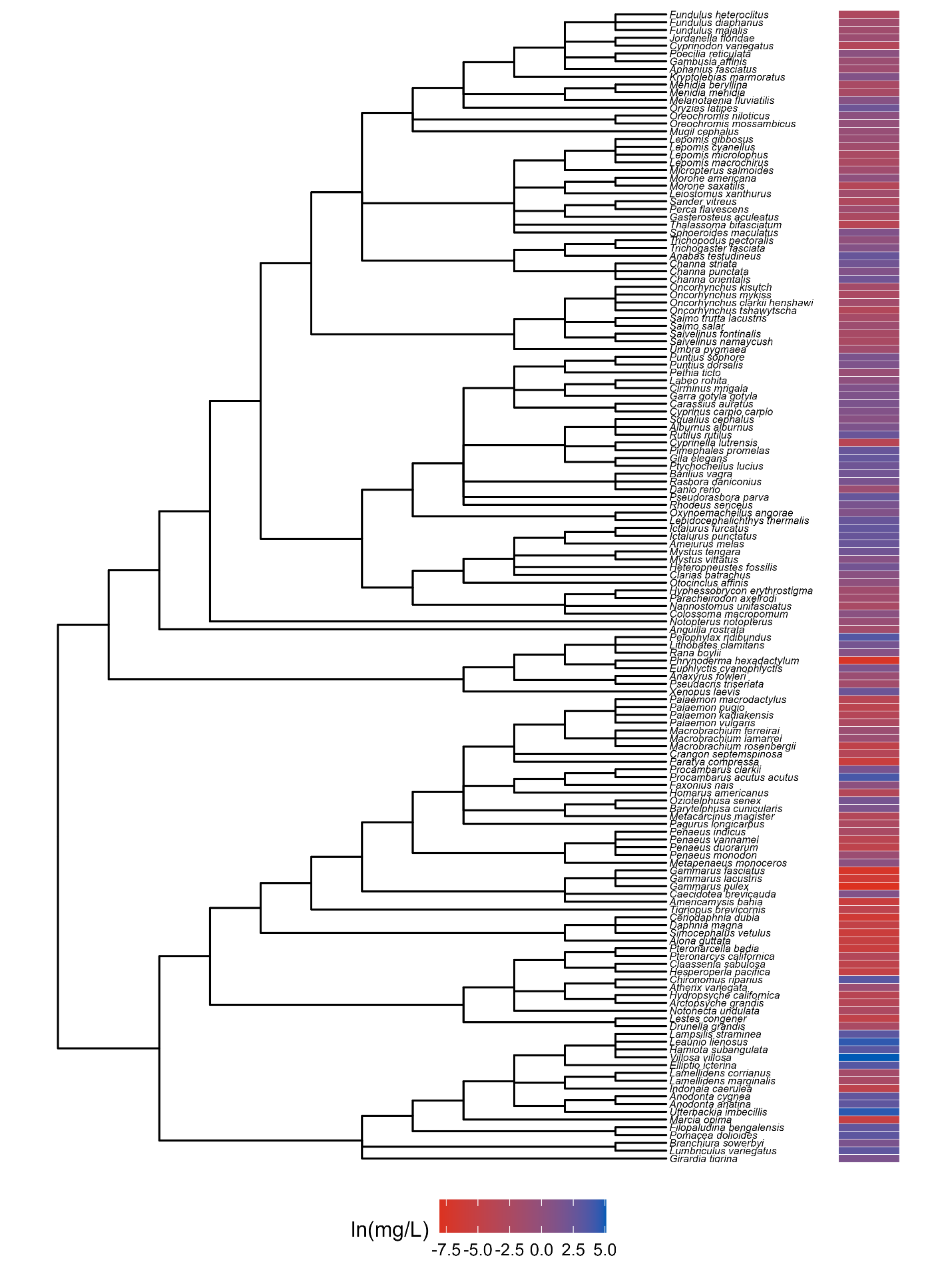
**

**Fig. S16** Phylogenetic tree and toxicity data heatmap for the complete acute malathion dataset (λ = 0.35). The colored bar next to each species represents its relative sensitivity to the chemical. A red bar indicates a high degree of sensitivity (i.e. small amount of chemical causes toxic effect), while a blue bar indicates low sensitivity (i.e. large amount of chemical causes toxic effect).

**
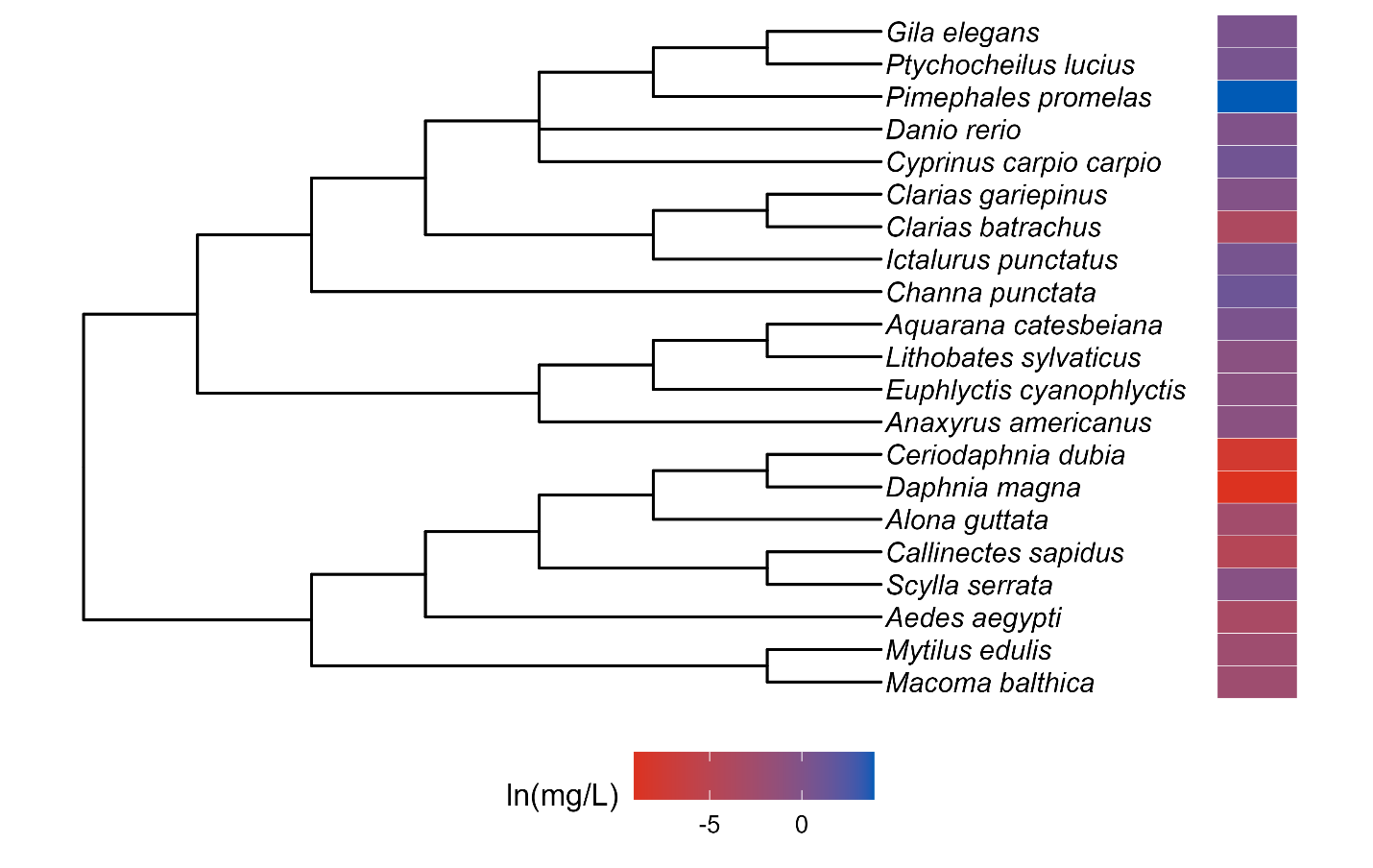
**

**Fig. S17** Phylogenetic tree and toxicity data heatmap for the complete chronic malathion dataset (λ = 7.3E-05). The colored bar next to each species represents its relative sensitivity to the chemical. A red bar indicates a high degree of sensitivity (i.e. small amount of chemical causes toxic effect), while a blue bar indicates low sensitivity (i.e. large amount of chemical causes toxic effect).


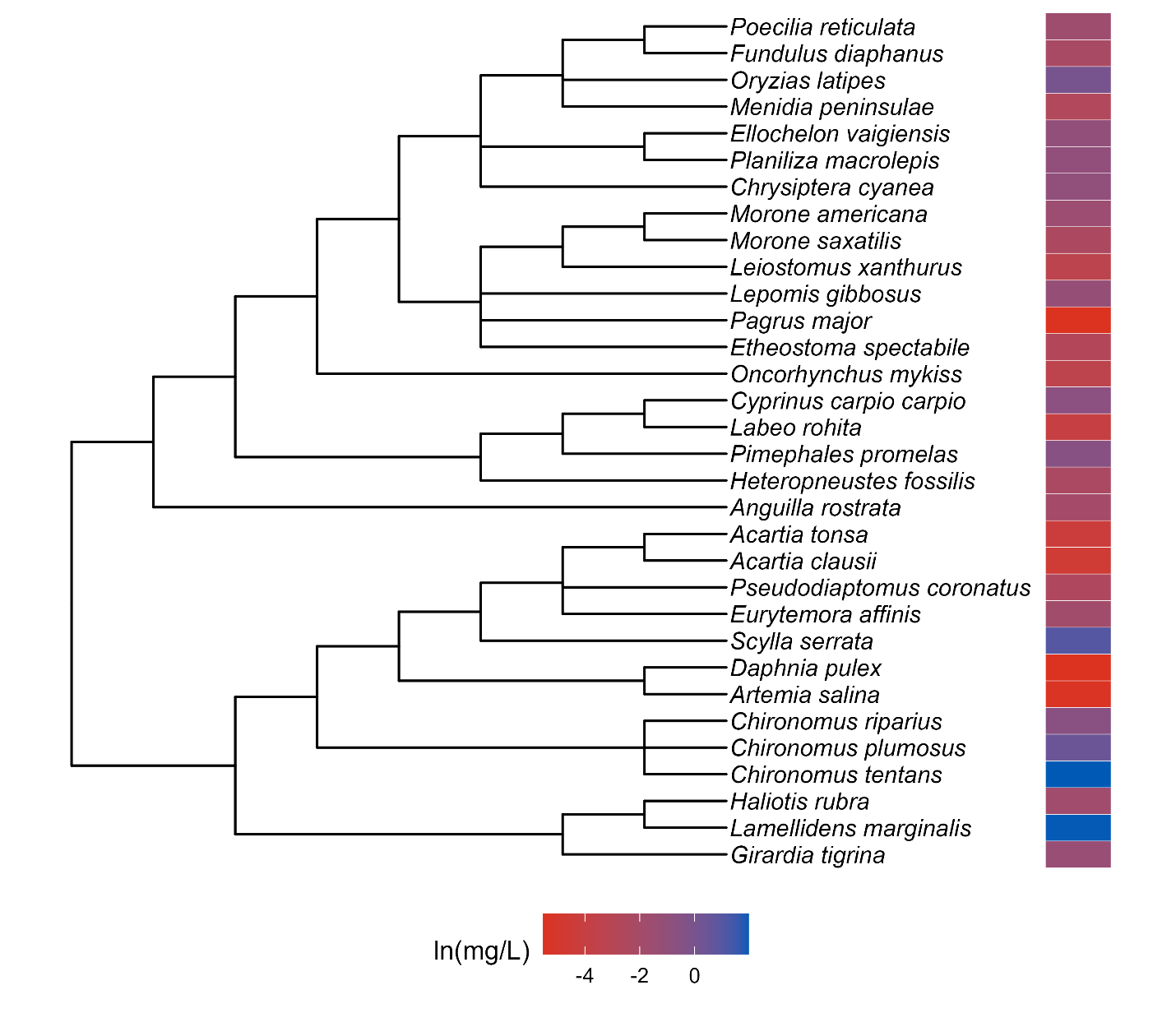


**Fig. S18** Phylogenetic tree and toxicity data heatmap for the complete acute mercury dataset (λ = 0.16). The colored bar next to each species represents its relative sensitivity to the chemical. A red bar indicates a high degree of sensitivity (i.e. small amount of chemical causes toxic effect), while a blue bar indicates low sensitivity (i.e. large amount of chemical causes toxic effect).


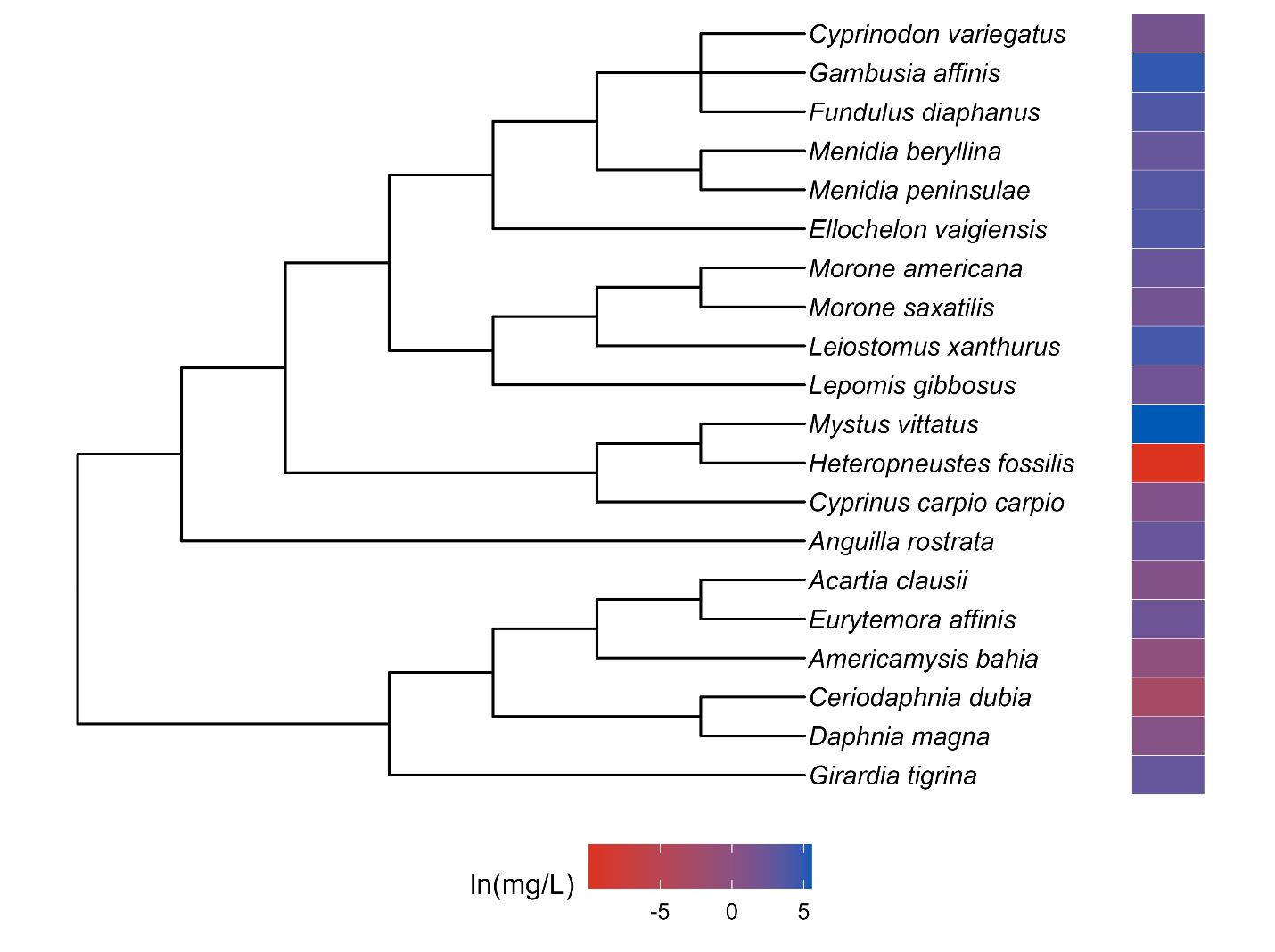


**Fig. S19** Phylogenetic tree and toxicity data heatmap for the complete acute nickel dataset (λ = 7.3E-05). The colored bar next to each species represents its relative sensitivity to the chemical. A red bar indicates a high degree of sensitivity (i.e. small amount of chemical causes toxic effect), while a blue bar indicates low sensitivity (i.e. large amount of chemical causes toxic effect).


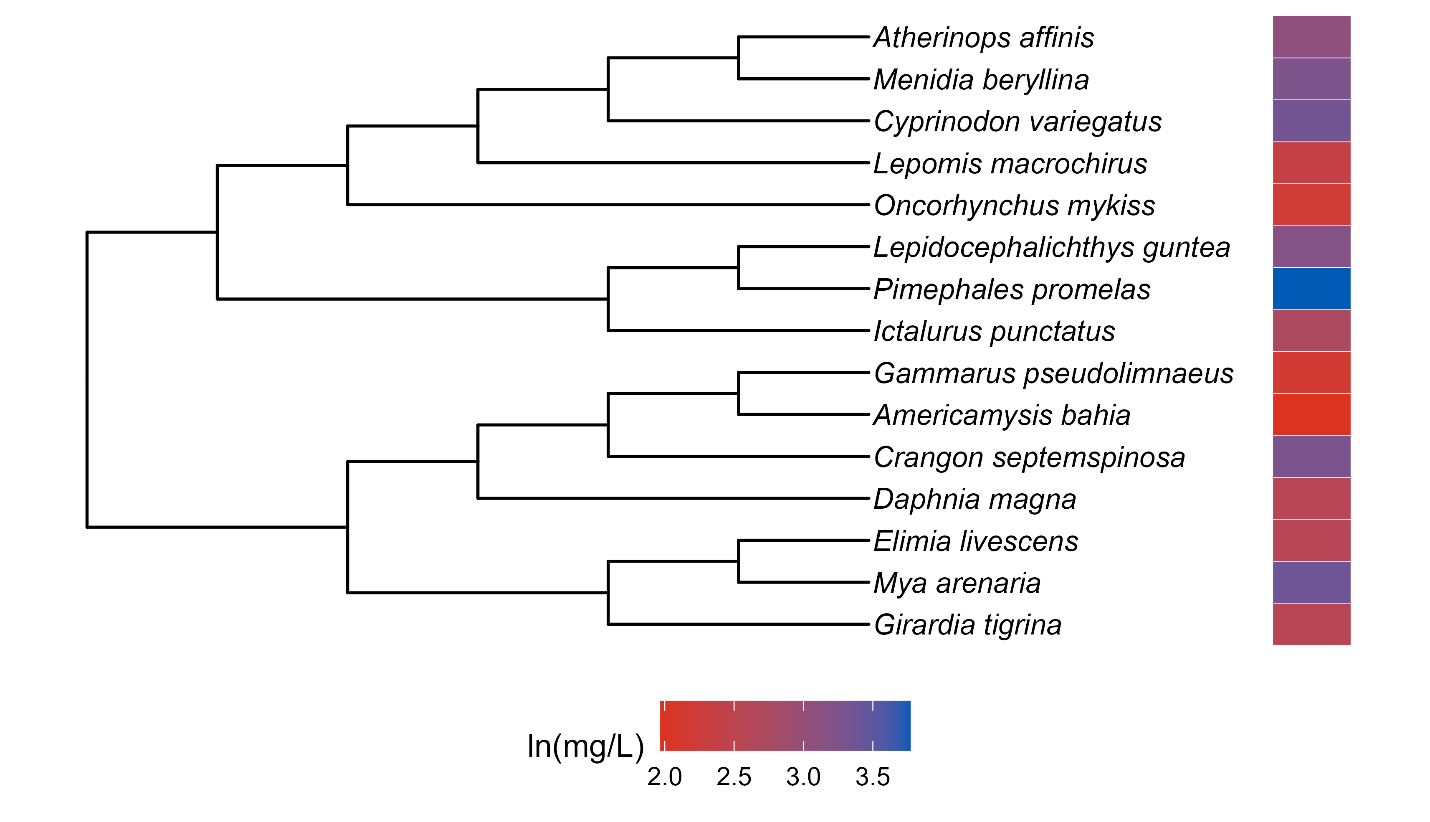


**Fig. S20** Phylogenetic tree and toxicity data heatmap for the complete acute 4-nitrophenol dataset (λ = 7.4E-05). The colored bar next to each species represents its relative sensitivity to the chemical. A red bar indicates a high degree of sensitivity (i.e. small amount of chemical causes toxic effect), while a blue bar indicates low sensitivity (i.e. large amount of chemical causes toxic effect).


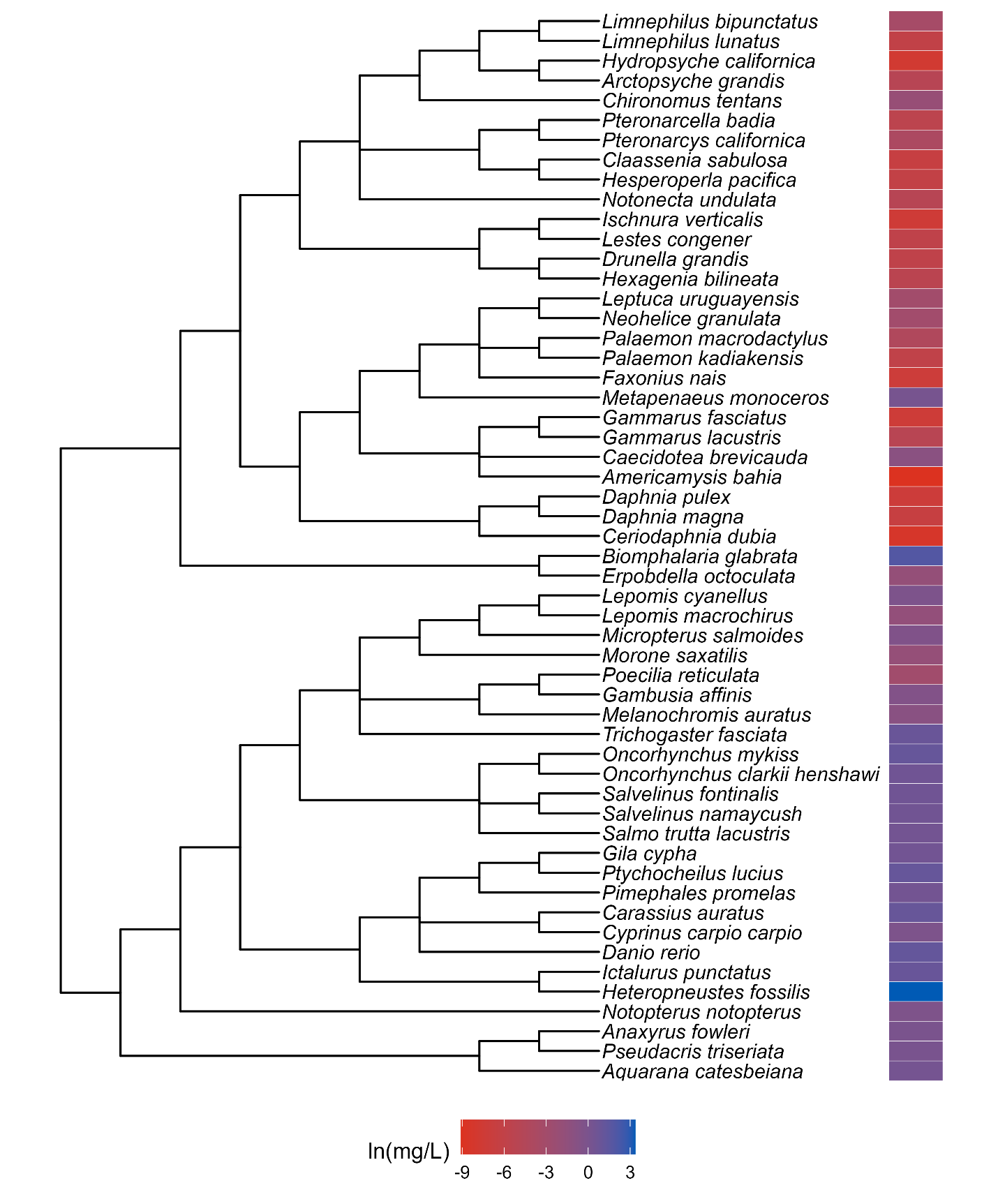


**Fig. S21** Phylogenetic tree and toxicity data heatmap for the complete acute parathion dataset (λ = 0.16). The colored bar next to each species represents its relative sensitivity to the chemical. A red bar indicates a high degree of sensitivity (i.e. small amount of chemical causes toxic effect), while a blue bar indicates low sensitivity (i.e. large amount of chemical causes toxic effect).


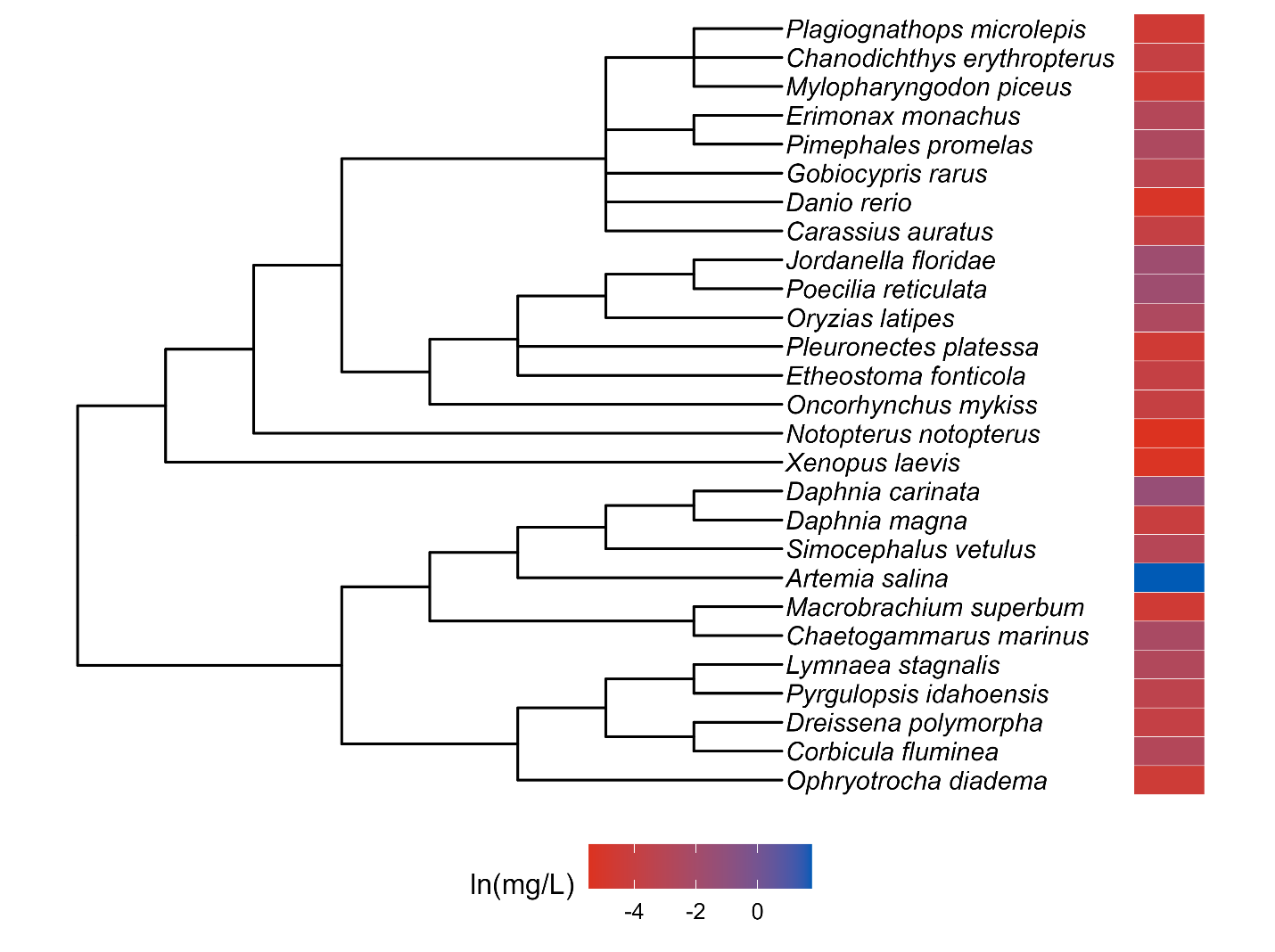


**Fig. S22** Phylogenetic tree and toxicity data heatmap for the complete chronic pentachlorophenol dataset (λ = 7.4E-05). The colored bar next to each species represents its relative sensitivity to the chemical. A red bar indicates a high degree of sensitivity (i.e. small amount of chemical causes toxic effect), while a blue bar indicates low sensitivity (i.e. large amount of chemical causes toxic effect).


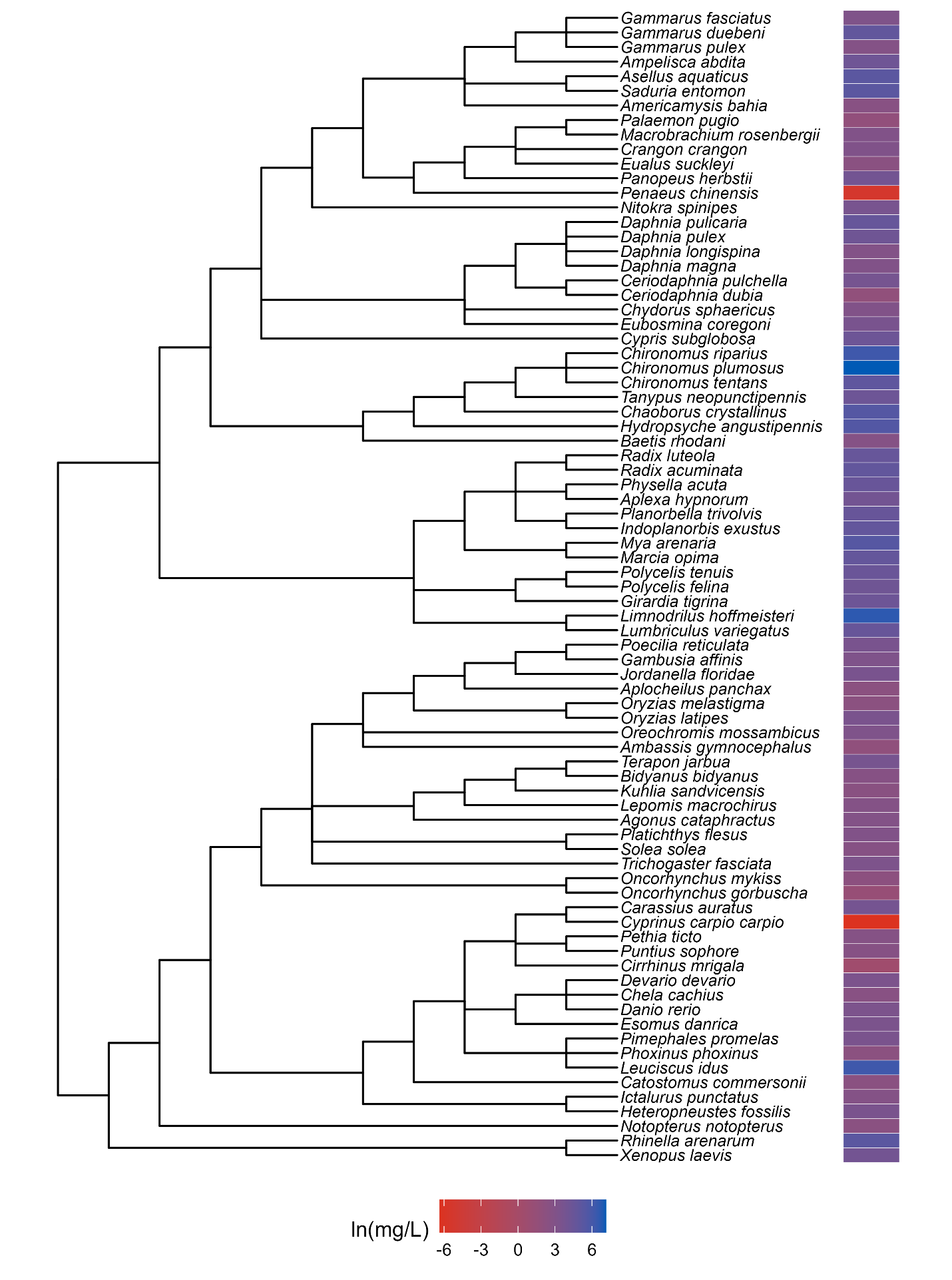


**Fig. S23** Phylogenetic tree and toxicity data heatmap for the complete acute phenol dataset (λ = 0.33). The colored bar next to each species represents its relative sensitivity to the chemical. A red bar indicates a high degree of sensitivity (i.e. small amount of chemical causes toxic effect), while a blue bar indicates low sensitivity (i.e. large amount of chemical causes toxic effect).


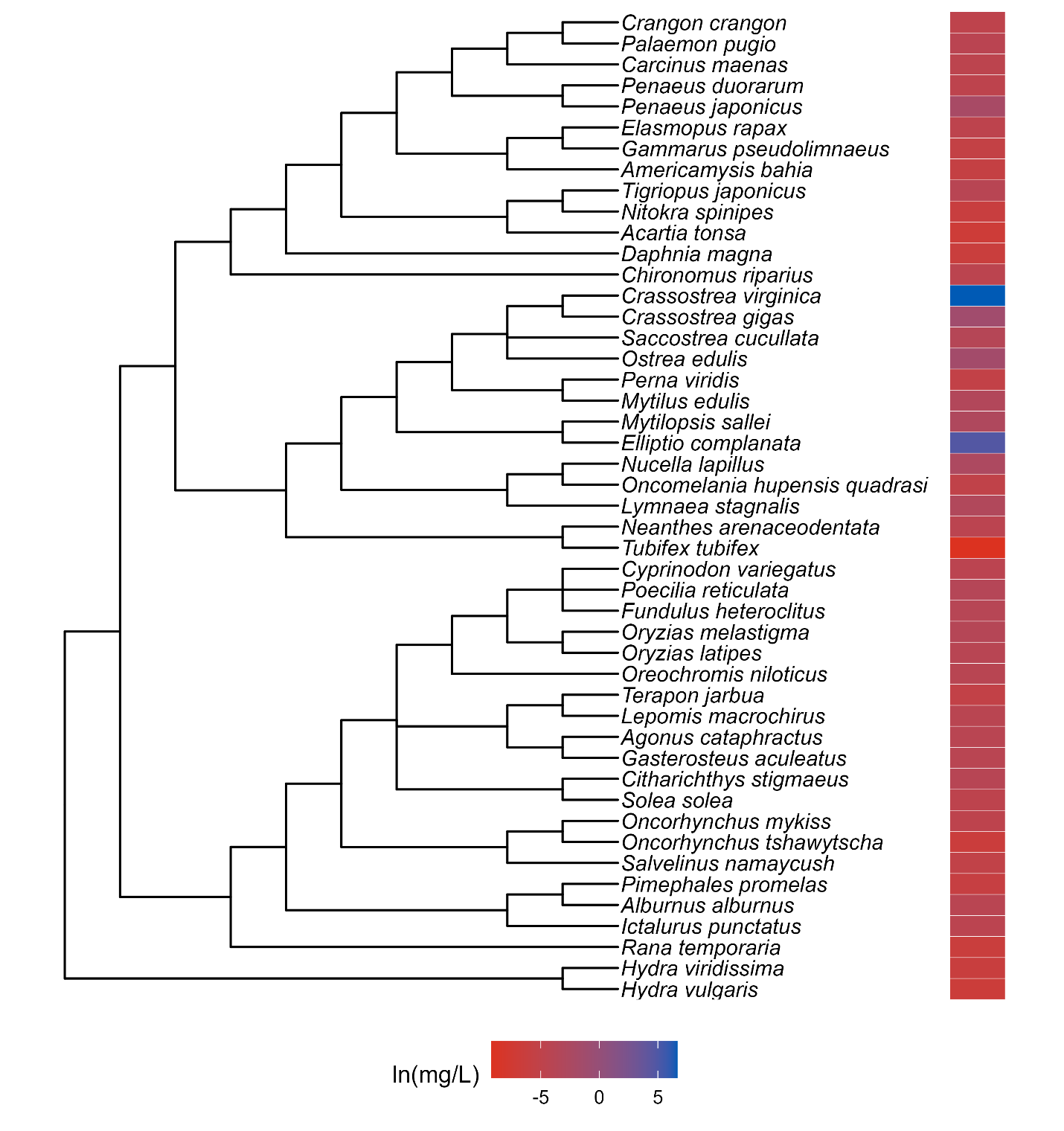


**Fig. S24** Phylogenetic tree and toxicity data heatmap for the complete acute TBTO dataset (λ = 7.3E-05). The colored bar next to each species represents its relative sensitivity to the chemical. A red bar indicates a high degree of sensitivity (i.e. small amount of chemical causes toxic effect), while a blue bar indicates low sensitivity (i.e. large amount of chemical causes toxic effect). (TBTO = Tributyltin oxide)


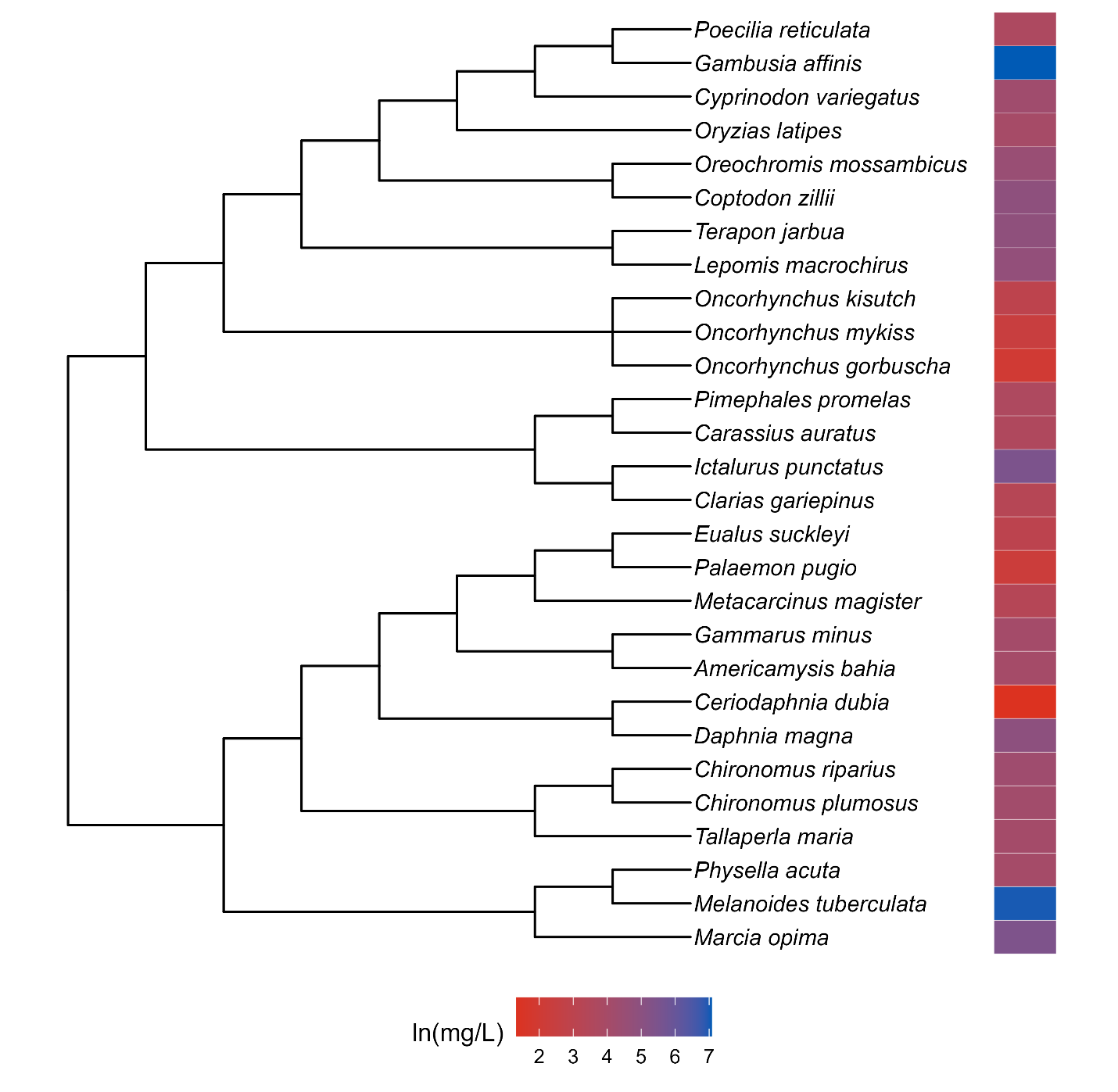


**Fig. S25** Phylogenetic tree and toxicity data heatmap for the complete acute toluene dataset (λ = 7.4E-05). The colored bar next to each species represents its relative sensitivity to the chemical. A red bar indicates a high degree of sensitivity (i.e. small amount of chemical causes toxic effect), while a blue bar indicates low sensitivity (i.e. large amount of chemical causes toxic effect).


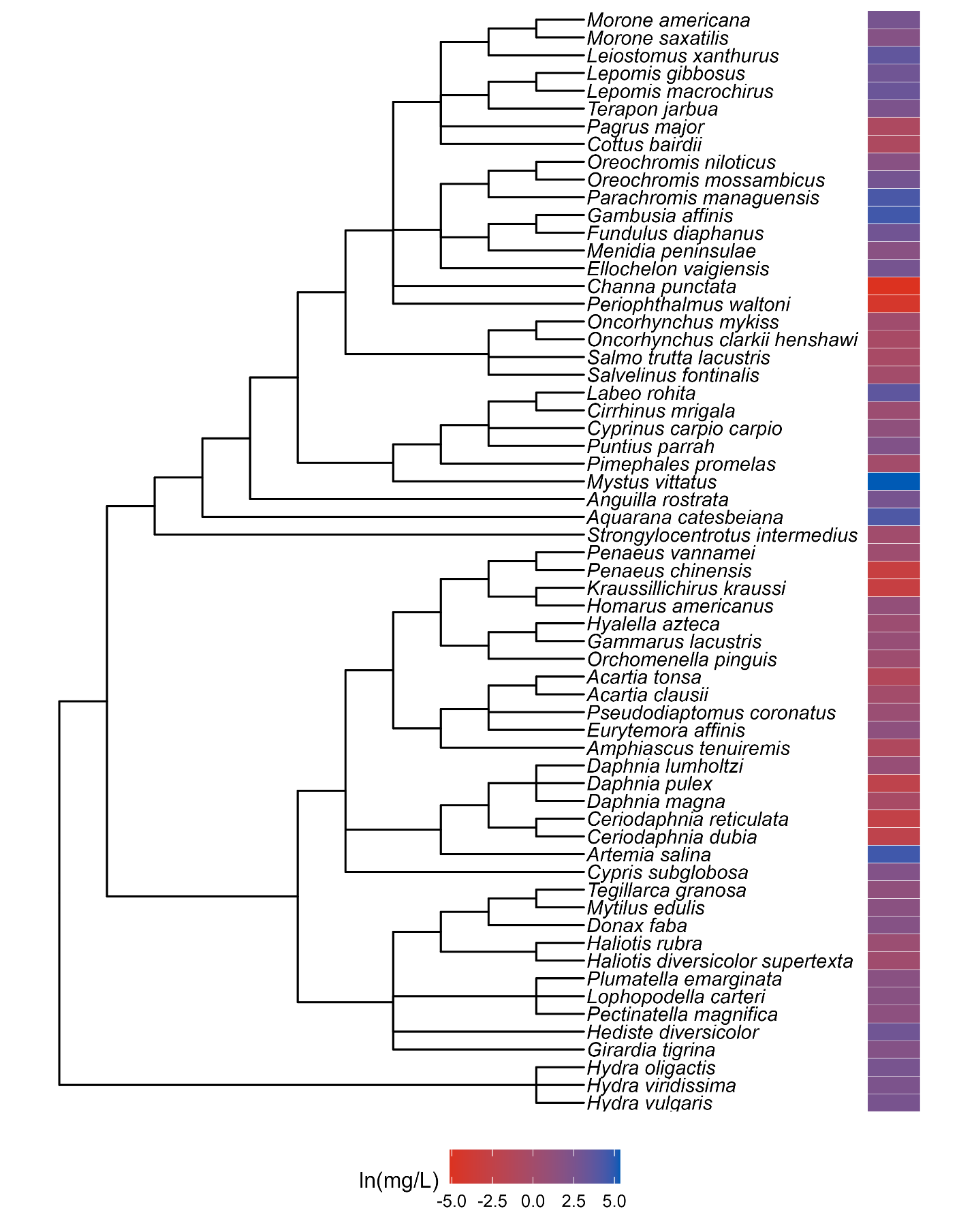


**Fig. S26** Phylogenetic tree and toxicity data heatmap for the complete acute zinc dataset (λ = 0.033). The colored bar next to each species represents its relative sensitivity to the chemical. A red bar indicates a high degree of sensitivity (i.e. small amount of chemical causes toxic effect), while a blue bar indicates low sensitivity (i.e. large amount of chemical causes toxic effect).


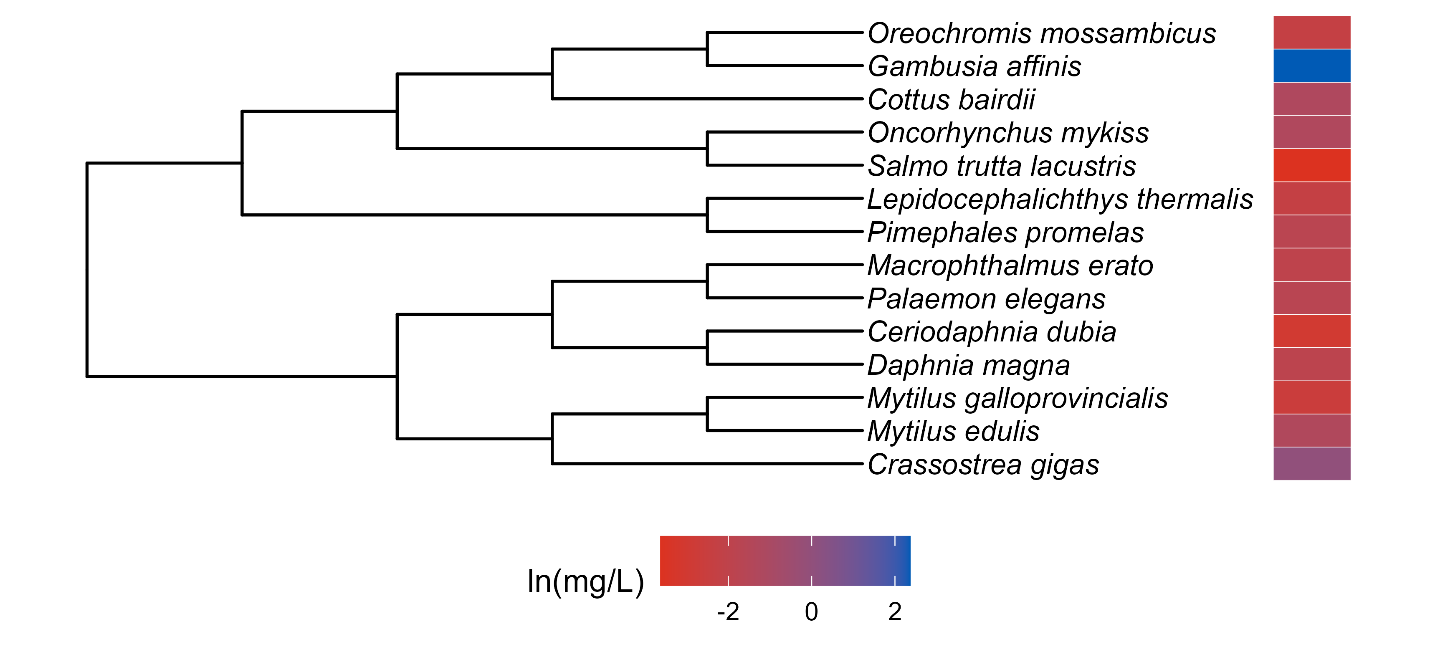


**Fig. S27** Phylogenetic tree and toxicity data heatmap for the complete chronic zinc dataset (λ = 7.3E-05). The colored bar next to each species represents its relative sensitivity to the chemical. A red bar indicates a high degree of sensitivity (i.e. small amount of chemical causes toxic effect), while a blue bar indicates low sensitivity (i.e. large amount of chemical causes toxic effect).
